# Association Between High-Density Lipoprotein Characteristics and Hemostatic Parameters in the Netherlands Epidemiology of Obesity (NEO) Study—Brief Report

**DOI:** 10.1161/ATVBAHA.125.323515

**Published:** 2026-01-21

**Authors:** Lushun Yuan, Jihee Han, Shuzhen Cheng, Frits R. Rosendaal, Dennis O. Mook-Kanamori, J. Wouter Jukema, Hans Vink, Bernard M. van den Berg, Ton J. Rabelink, Astrid van Hylckama Vlieg, Uwe J.F. Tietge, Ko Willems van Dijk, Ruifang Li-Gao

**Affiliations:** 1 Department of Vascular Surgery, Intervention Center, Shanghai General Hospital, Shanghai Jiao Tong University School of Medicine, People’s Republic of China (L.Y.).; 2 Einthoven Laboratory for Vascular and Regenerative Medicine, Department of Internal Medicine, Nephrology (L.Y., B.M.v.d.B., T.J.R.), Leiden University Medical Center, the Netherlands.; 3 Department of Clinical Epidemiology (J.H., F.R.R., D.O.M.-K., A.v.H.V., R.L.-G.), Leiden University Medical Center, the Netherlands.; 4 Department of Public Health and Primary Care (D.O.M.-K.), Leiden University Medical Center, the Netherlands.; 5 Department of Cardiology (J.W.J.), Leiden University Medical Center, the Netherlands.; 6 Department of Human Genetics (K.W.v.D.), Leiden University Medical Center, the Netherlands.; 7 Division of Endocrinology, Department of Internal Medicine (K.W.v.D.), Leiden University Medical Center, the Netherlands.; 8 Leiden Laboratory for Experimental Vascular Medicine (K.W.v.D.), Leiden University Medical Center, the Netherlands.; 9 SKL of Marine Food Processing and Safety Control, School of Food Science and Technology (S.C.), Dalian Polytechnic University, People’s Republic of China.; 10 National Engineering Research Center of Seafood, Collaborative Innovation Center of Seafood Deep Processing (S.C.), Dalian Polytechnic University, People’s Republic of China.; 11 Netherlands Heart Institute, Utrecht (J.W.J.).; 12 Glycocalyx Research Institute, Alpine, UT (H.V.).; 13 Division of Clinical Chemistry, Department of Laboratory Medicine, Karolinska Institutet, Stockholm, Sweden (U.J.F.T.).; 14 Clinical Chemistry, Karolinska University Laboratory, Karolinska University Hospital, Stockholm, Sweden (U.J.F.T.).

**Keywords:** antioxidants, cardiovascular diseases, thrombin, triglycerides, venous thromboembolism

## Abstract

**BACKGROUND:**

Recent evidence points to connections between HDLs (high-density lipoproteins), the coagulation system, and venous thromboembolism occurrence. However, uncertainty remains regarding the impact of specific HDL characteristics on the coagulation system. This study investigated associations between HDL characteristics and hemostatic parameters in a large middle-aged Dutch population.

**METHODS:**

Using baseline measurements from 6245 participants in NEO study (the Netherlands Epidemiology of Obesity), we performed adjusted linear regression analyses to estimate associations between 34 parameters of XLHDL (very large HDL), LHDL (large HDL), MHDL (medium HDL), and SHDL (small HDL) particles, as well as ApoA1 (apolipoprotein A1), quantified using a high-throughput ^1^H-nuclear magnetic resonance metabolomics platform, and coagulation parameters. These included coagulation factor (F) VIII, FIX, FXI, and fibrinogen, along with 5 parameters of the thrombin generation potential. In addition, the associations between HDL characteristics and parameters of platelet activation and endothelial glycocalyx health were tested in a subpopulation.

**RESULTS:**

Our findings revealed a particle size–dependent association between HDL parameters and coagulation parameters. Particularly, per 1-SD increase in the levels of components within XLHDL (very large HDL), we observed lower levels in FIX and FXI activities, endogenous thrombin potential, and peak height (median β [interquartile range], FIX: 3.26% [−3.50% to −3.18%]; FXI: −0.96% [−1.21% to −0.89%]; endogenous thrombin potential: −22.11 [−27.07 to −21.47] nmol/L·min; and peak height: −2.28 [−2.70 to −2.19] nmol/L), indicating an antithrombotic effect. In contrast, per 1-SD increase in the levels of components within MHDL and SHDL, we observed an increase in endogenous thrombin potential, peak height, and activities of FVIII, FIX, and FXI, indicating a prothrombotic effect. HDL characteristics were not associated with platelet activation parameters or with glycocalyx-related parameters.

**CONCLUSIONS:**

Our study provides evidence for a size-dependent relationship between HDL components and coagulation parameters. These findings contribute to a better understanding of the potential role of HDL in the pathogenesis of venous thromboembolism.

**What Are the Clinical Implications?**

Recent evidence from the UK Biobank shows that larger HDL (high-density lipoprotein) particle size is associated with a lower risk of venous thromboembolism, while smaller HDL particles confer a higher risk, demonstrating a clear dose-response relationship across the HDL size spectrum. In this study, we further observed a particle size–dependent association between HDL components and hemostatic parameters: very large and large HDL components were linked to lower levels of procoagulant factors and a reduced thrombin generation potential, whereas medium and small HDL components showed the opposite pattern. Together, these findings suggest that HDL particle profiling, beyond traditional HDL-cholesterol measurement, may offer additional value in identifying individuals at higher thrombotic risk and could potentially guide more personalized preventive strategies in clinical practice.

The link between obesity and an increased risk of venous thromboembolism (VTE) involves multifactorial pathways, including disturbed lipid homeostasis.^1^ While lipid measurements (ie, total cholesterol, LDL [low-density lipoprotein] cholesterol, HDL [high-density lipoprotein] cholesterol, or triglycerides) have shown inconsistent associations with VTE risk,^2–4^ a meta-analysis has reported lower HDL-cholesterol levels in patients with VTE.^5^ Notably, high HDL cholesterol and its component ApoA1 (apolipoprotein A1) demonstrate protective effects against VTE.^2,6,7^ Recent evidence from the UK Biobank further indicates that larger HDL particle size is associated with a reduced risk of VTE, whereas smaller HDL particle size increases the risk, showing a clear dose-response pattern across the HDL size spectrum.^8^ However, this study did not explore potential underlying mechanisms, highlighting the need for further research into the hemostatic system.

HDL particles are heterogeneous in size, composition, and function.^9–13^ SHDL (small HDL) particles are thought to be acceptors of cholesterol from peripheral sources, whereas LHDL (large HDL) particles are thought to deliver cholesterol to the liver for clearance.^14^ Although total HDL cholesterol is inversely associated with cardiovascular disease risk, Mendelian randomization studies indicate that this association is likely not causal.^15^ Nevertheless, cholesterol efflux from cholesterol laden cells to HDL is more strongly associated with cardiovascular disease risk than total HDL cholesterol.^16^ In addition, LHDL particles have also been shown to have anti-inflammatory and antioxidant properties.^17^ Therefore, specific HDL particles may have differential effects on cardiovascular and other disease outcomes. Whether specific HDL characteristics affect the hemostatic system, a central mechanism in VTE development, remains unclear.

Our study addresses this gap by investigating the association between HDL characteristics, assessed by high-throughput ^1^H-nuclear magnetic resonance metabolomics and a broad panel of directly measured hemostatic factors, including coagulation factors, thrombin generation parameters, and platelet activation parameters in a large, middle-aged Dutch population, NEO study (the Netherlands Epidemiology of Obesity). Furthermore, we sought to elucidate the potential involvement of the endothelial glycocalyx, a marker of endothelial health and coagulation balance,^18,19^ as a mediator in the association between HDL characteristics and hemostatic factors. By integrating detailed HDL profiling with functional measures of coagulation, our study provides mechanistic insight that complements and extends recent findings from the UK Biobank, helping to clarify the role of HDL in VTE risk.

## Methods

The data that support the findings of this study are available from the corresponding author upon request.

### Study Population

This study is a cross-sectional analysis of baseline data (collected between 2008 and 2012) from the NEO study, a population-based cohort comprising 6671 participants aged 45 to 65 years.^20^ All participants gave their written informed consent. The NEO study was approved by the Medical Ethics Committee of the Leiden University Medical Center, Leiden, the Netherlands (P08.109). During the baseline visit, blood samples were taken after an overnight fast of at least 10 hours. After exclusion of participants with (1) missing values of HDL characteristics data (n=103), (2) missing values of outcomes (ie, coagulation factors and thrombin generation parameters; n=173), (3) use of anticoagulant therapy at the time of venipuncture (ie, vitamin K antagonists or heparin; n=130), (4) missing values of confounding factors (n=39), and (5) extreme values (*Z* score >5) in outcomes (ie, coagulation factors and thrombin generation parameters; n=72), 6245 participants were included in the analyses with coagulation parameters as outcome variables (Figure S1). The same exclusion criteria were applied to include individuals in the investigation of the associations between HDL characteristics and platelet activation parameters (Figure S2; n=1697) and to estimate the potential mediating effects of endothelial glycocalyx function on the association between HDL characteristics and hemostatic parameters (Figure S3; n=824).

### Measurements of HDL Characteristics

HDL characteristics were measured using high-throughput ^1^H-nuclear Nightingale metabolomics, which quantified 229 metabolites and metabolite ratios.^21^ The current analyses included ApoA1 concentration, triglyceride concentration in total HDL, cholesterol concentration in total HDL, HDL2, and HDL3, and average HDL particle diameters, as well as HDL particle number and lipid content (ie, total lipid, total cholesterol, free cholesterol, cholesterol ester, phospholipid, and triglyceride) in 4 HDL subclasses (XLHDL [very LHDL]: 14.3 nm; LHDL: 12.1 nm; MHDL [medium HDL]: 10.9 nm; and SHDL: 8.7 nm). To be noted, HDL2 and HDL3 concentrations were quantified using the Nightingale Health NMR platform, where these subclasses are defined according to particle size distributions estimated from NMR spectra (HDL2=larger particles; HDL3=smaller particles) and do not directly correspond to the classical density-based HDL2/HDL3 fractions from ultracentrifugation. Detailed information, including quality-assurance measures and applications of the platform, has been given elsewhere.^21^ Detailed abbreviations of HDL characteristics are shown in Table S1.

### Measurements of Hemostatic Factors

The activities of coagulation factors VIII, IX, and XI, along with fibrinogen levels, thrombin generation potential, and platelet activation parameters^22^ were measured as the outcomes. Fibrinogen levels were measured using the Clauss method. The activities of coagulation factor (F) VIII, FIX, and FXI were measured in activity assays with a coagulometric clot detection method on an ACL TOP 700 analyzer (Werfen, Barcelona, Spain). The measurements were calibrated using Werfen plasma as reference plasma. The activities of FVIII, FIX, and FXI were reported as percentages (%) of pooled normal plasma levels. Fibrinogen levels were expressed in mg/dL.

Thrombin generation was measured following the protocols described by Hemker et al^23^: calibrated automated thrombogram (Thrombinoscope BV, Maastricht, the Netherlands). In brief, 20 μL of PPP-Reagent LOW (86194, TS31.00, STAGO, France) and thrombin calibrator (86192, TS30.00, STAGO) were added to the wells of a round-bottom 96-well plate (3655, Thermo Scientific, Uden, the Netherlands). Plasma samples from participants in the NEO study and normal pooled plasma as an internal control for each plate were supplemented with TICA (thermostable inhibitor of contact activation; PS-0177-oxoxox, Maastricht, the Netherlands). The plate was then filled with 80 μL of mixed plasma and incubated in a fluorometer at 37 °C for 10 minutes. Thrombin formation was initiated by adding 20 μL of the fluorogenic substrate along with calcium (FluCa-kit, 86197, TS 50.00, STAGO). The final reaction volume was 120 μL. Thrombin formation was measured every 10 seconds for 50 minutes and calibrated using the Thrombinoscope software. Various parameters were determined for thrombin generation, including lag time, time-to-peak, peak height, endogenous thrombin potential (ETP), and velocity. Lag time and time-to-peak were reported in minutes (min), peak height in nM, ETP in nM·min, and velocity in nM/min. A shorter lag time and time-to-peak, higher peak height, larger ETP, and increased velocity suggest hypercoagulability. Because ETP and peak height consistently showed associations with VTE risk, we focused on these 2 parameters when reporting the results.

Platelet count, mean platelet volume, and platelet distribution width were determined in a random subset of participants in the central clinical hematology laboratory of the Leiden University Medical Center via hydrodynamic focusing (DC detection) or a flow cytometry method with semiconductor laser.

### Sidestream Dark Field Microvascular Imaging for Glycocalyx Measurement

In a subpopulation of the NEO study, sidestream dark field microscopy (MicroVision Medical, Inc, Wallingford, PA) was performed on individuals in a supine position. Data were acquired and analyzed using GlycoCheck software (Glycocalyx Research Institute, Alpine, UT). The software automatically identifies all available perfused microvessels distributed at a 1-µm interval between 4 and 25 µm, and red blood cell velocity was included as a new parameter with newly developed software.^24^ After reanalysis, the following validated glycocalyx-related parameters were included in this study: total vessel perfused boundary region (PBR_Total_, 4–25 µm), PBR feed vessel (PBR_feed vessel_, 10–19 µm), and PBR capillary (PBR_capillary_, 4–9 µm).

### Confounding Factors

In the baseline questionnaire, participants reported age, sex, and family and personal medical history. Self-identified race was reported in 8 categories, which were grouped into White and others. Menopausal status was classified as premenopausal and perimenopausal or postmenopausal, according to information on oophorectomy, hysterectomy, and self-reported state of menopause in the questionnaire. Height was measured with a vertically fixed, calibrated tape measure. Body weight and percent body fat were measured by the Tanita bioimpedance balance (International Division, United Kingdom) without shoes, and 1 kg was subtracted to correct for the weight of clothing. The body mass index was calculated by dividing the weight in kilograms by the height in meters squared. The serum concentrations of CRP (C-reactive protein) were determined using a high-sensitivity CRP assay (TINA-Quant CRP HS system and Modular P800, Roche).

### Statistical Analyses

For the analyzing the associations between HDL parameters and hemostatic factors, analyses were weighted to account for body mass index oversampling, ensuring general population representativeness. We examined distributions of confounders and HDL parameters (z-transformed), applied log transformations where needed, and standardized hemostatic outcomes for consistent interpretation. Linear regression was used to assess associations between HDL parameters and hemostatic factors across progressively adjusted models, with false discovery rate correction applied. Additional analyses included sex-stratified models and exploratory mediation analyses to assess the role of glycocalyx perturbation. Detailed descriptions can be found in Supplemental Methods.

## Results

### Participant Characteristics

Table S2 summarizes baseline characteristics. The median age of the 6245 participants was 57 (interquartile range, 51–61) years, 56% were women, and 81.7% of them were perimenopausal or postmenopausal. The weighted median body mass index was 24.6 (interquartile range, 22.9–26.2) kg/m², and median total body fat was 30.2% (interquartile range, 22.9%–36.5%). Lipid-lowering medications were used by 10.2% of participants. The median values of coagulation, platelet activation, and glycocalyx-related parameters are summarized in Table S2, while Tables S3 and S4 present baseline characteristics for the subpopulations used in the platelet activation and glycocalyx analyses.

### Associations Between HDL Characteristics and Coagulation, as well as Thrombin Generation Parameters

**Figure 1.**
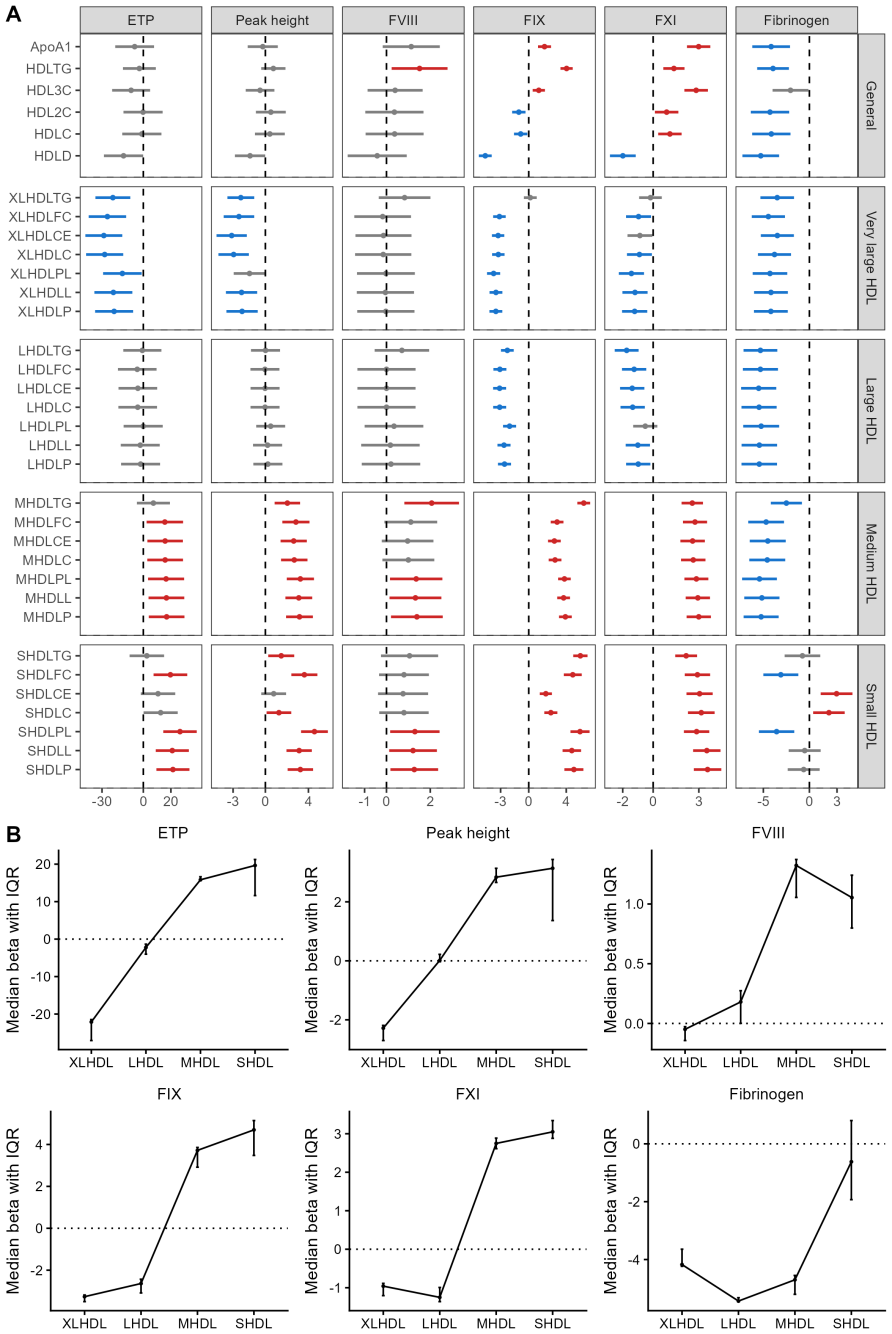
**Associations between HDL (high-density lipoprotein) characteristics and levels of coagulation parameters including endogenous thrombin potential (ETP), peak height, factor (F) VIII, FIX, FXI, and fibrinogen in the total population. A**, After adjustment for age, sex, race, menopausal status, lipid-lowering drugs, C-reactive protein, total body fat, and body mass index, differences in the total population between HDL characteristics and levels of coagulation parameters were observed. The effect size with a 95% CI was depicted by a dot with a horizontal line. After multiple testing correction, nonsignificant associations were shown in gray, significant positive associations in red, and significant negative associations in blue. **B**, Particle size–dependent differences in the total population between HDL components and levels of coagulation parameters were illustrated. The median effect size and interquartile range of HDL components (ie, particle number and lipid content including total lipid, total cholesterol, free cholesterol, cholesterol ester, phospholipid, and triglyceride) for each particle size were shown. Detailed abbreviations of HDL characteristics are shown in Table S1. ApoA1 indicates apolipoprotein A1; HDL2C, total cholesterol in HDL2; HDL3C, total cholesterol in HDL3; HDLC, total cholesterol in HDL; HDLCE, cholesteryl esters in HDL; HDLD, average diameter for HDL particles; HDLFC, free cholesterol in HDL; HDLL, total lipids in HDL; HDLP, concentration of HDL particles; HDLPL, phospholipids in HDL; HDLTG, total triglycerides in HDL; LHDL, large HDL; MHDL, medium HDL; SHDL, small HDL; and XLHDL, very large HDL.

We first focused on characteristics of all HDL particles combined, which included ApoA1 concentration, triglyceride and cholesterol concentration in total HDL (ie, total triglycerides in HDL, total cholesterol in HDL, total cholesterol in HDL2, and total cholesterol in HDL3), and average HDL particle diameter (ie, average diameter for HDL particles). In models 1 and 2, HDL parameters were negatively associated with ETP, peak height, velocity, FIX activity, and fibrinogen but positively with FXI activity, lag time, and time-to-peak. Triglycerides in total HDL showed opposite associations (Tables S5 and S6). In the fully adjusted model 3, average HDL particle diameter remained negatively associated with FIX, FXI, and fibrinogen while showing positive associations with FXI activity (Figure 1A), lag time, and time-to-peak (Table S7).

Because the average diameter of HDL particles exhibited negative associations with coagulation parameters in model 3, we examined the associations between the quantity of HDL particles of varying sizes and coagulation parameters (Figure 1A). A higher particle number of XLHDL was associated with lower ETP (−21.20 [95% CI, −35.00 to −7.41] nmol/L·min), peak height (−2.16 [95% CI, −3.62 to −0.71] nmol/L), FIX (−3.51% [95% CI, −4.19% to −2.82%]), and FXI (−1.22% [95% CI, −2.04% to −0.39%]) activities, whereas a higher particle number of MHDL and SHDL was linked to higher ETP (MHDL: 16.91 [95% CI, 3.98–29.85] nmol/L·min; SHDL: 21.48 [95% CI, 9.49–33.47] nmol/L·min), peak height (MHDL: 3.17 [95% CI, 1.92–4.41] nmol/L; SHDL: 3.25 [95% CI, 2.07–4.43] nmol/L), FIX (MHDL: 3.91% [95% CI, 3.23%–4.59%]; SHDL: 4.81% [95% CI, 3.80%–5.83%]), and FXI (MHDL: 3.00% [95% CI, 2.20%–3.79%]; SHDL: 3.57% [95% CI, 2.67%–4.48%]) activities. The particle numbers of XLHDL, LHDL, and MHDL were negatively associated with fibrinogen, while SHDL showed no association. Overall, size-specific results (Tables S5 through S7) were consistent with the general HDL findings.

HDL lipid components showed size-dependent associations with coagulation parameters (Figure 1B). XLHDL and LHDL exhibited anticoagulant effects, negatively associating with FIX, FXI, ETP, peak height, and fibrinogen, while MHDL and SHDL showed procoagulant effects, positively associating with FIX, FXI, FVIII, ETP, and peak height. Overall, these findings indicate a distinct size-dependent influence of HDL lipid components on coagulation.

### Associations Between HDL Characteristics and Platelet Activation Parameters

Tables S8 through S10 present the associations between HDL characteristics and platelet activation parameters. In model 1, HDL characteristics were positively associated with platelet count after multiple testing corrections, except for SHDL (Table S8). However, none of these findings remained significant after a further adjustment for confounders (Tables S9 and S10).

### Associations Between HDL Characteristics and Glycocalyx-Related Parameters

Tables S11 through S13 show the associations between HDL characteristics and levels of glycocalyx-related parameters. In model 1, LHDL and MHDL components, levels of ApoA1, total cholesterol in HDL and HDL2, and average HDL diameter were positively associated with PBR_feed vessel_ and PBR_Total_, while SHDL triglycerides were negatively associated. However, none of these associations remained significant after multiple testing correction (Table S13). Therefore, no further mediation analysis was performed (Tables S11 through S13).

### Stratification Analyses by Sex

**Figure 2.**
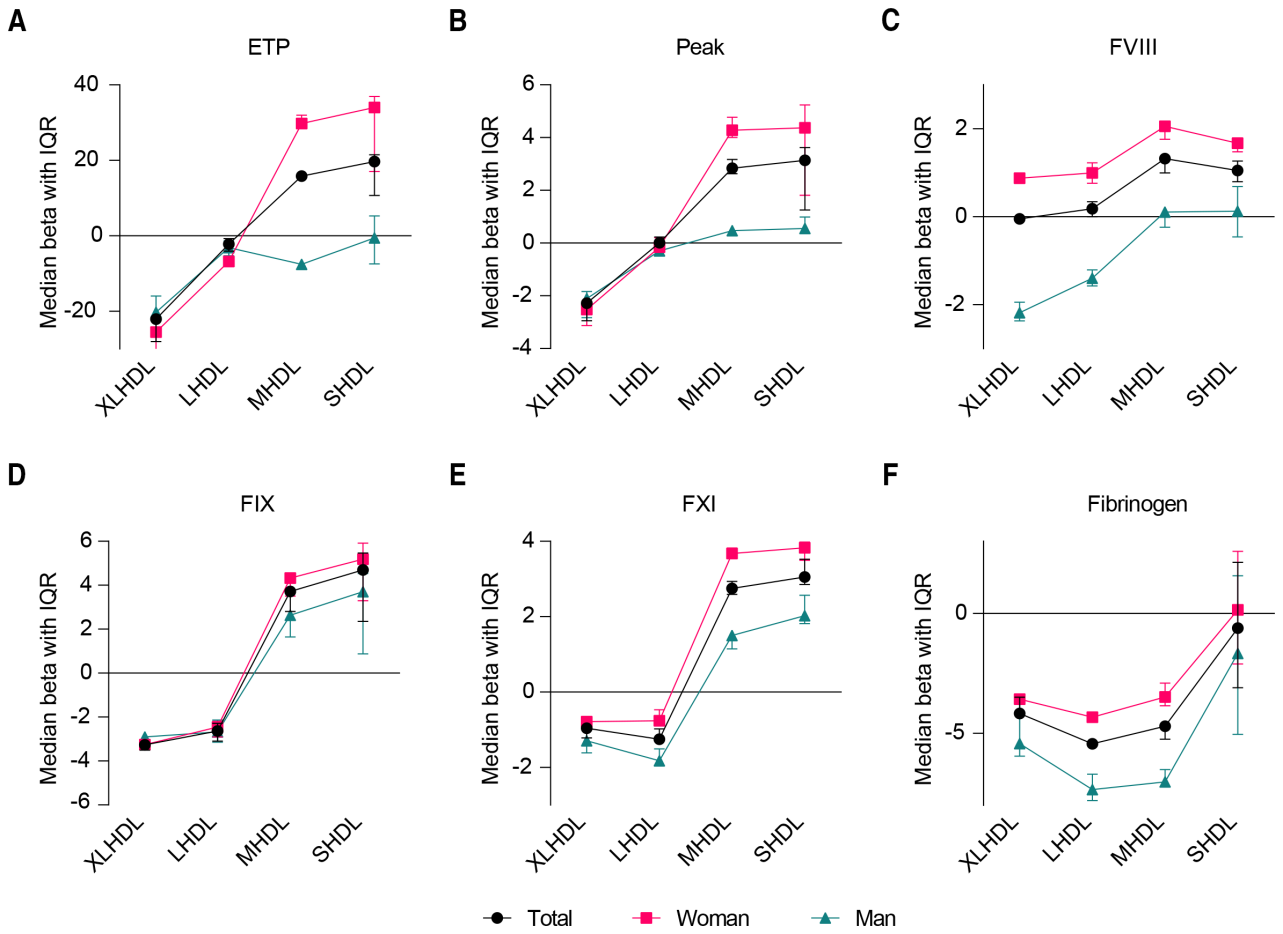
**Particle size–dependent differences between HDL (high-density lipoprotein) components and levels of coagulation parameters.** After adjustment for age, race, menopausal status, lipid-lowering drugs, C-reactive protein, total body fat, and body mass index, as well as sex for the total population, the median effect size and interquartile range of the associations between HDL components (ie, particle number and lipid content including total lipid, total cholesterol, free cholesterol, cholesterol ester, phospholipid, and triglyceride) and coagulation parameters (ie, endogenous thrombin potential [ETP], peak height, factor (F) VIII, FIX, FXI, and fibrinogen) for each particle size in men, women, and the total population were shown. The red line represents women, the green line represents men, and the black line represents the total population.

Size-dependent associations between HDL components and hemostatic parameters were present in both men and women (Figure 2; Tables S14 and S15), with notable sex differences. Specifically, women showed stronger positive associations of MHDL and SHDL components with ETP, peak height, FVIII, and FXI activities, whereas men exhibited stronger negative associations of XLHDL and LHDL components with FVIII, FXI, and fibrinogen. Figure S4 further illustrates these sex-specific differences, with women generally showing larger effect estimates.

## Discussion

This study examined associations between HDL characteristics and hemostatic factors, revealing particle size–dependent effects on coagulation. XLHDL and LHDL components were associated with lower levels of procoagulants and reduced thrombin generation potential, whereas MHDL and SHDL components showed the opposite pattern, with stronger associations in women. No associations were found with platelet activation or glycocalyx parameters, suggesting that the glycocalyx is unlikely to mediate HDL-coagulation relationships. These results provide functional evidence addressing critical mechanistic gaps highlighted in a previous publication from the UK biobank.^8^ Instead of considering HDL as a uniform entity, our results emphasize its heterogeneity and the distinct associations between particle subtypes and hemostatic parameters. By directly relating HDL particle size to coagulation factor activities and thrombin generation parameters, our study provides phenotypic evidence for potential mechanisms through which HDL may contribute to thrombotic risk. This functional perspective complements earlier observations and helps to better understand the role of HDL in VTE development.

Thrombin generation potential has been strongly associated with an increased VTE risk,^25,26^ which indicates that these parameters may serve as a valuable intermediate phenotype for VTE. Building on previous research, we found that a higher concentration of XLHDL components was associated with lower ETP, while a higher concentration of MHDL and SHDL components was associated with higher ETP. Similarly, it was found that HDL2 particles exhibited higher anticoagulant activity than HDL3 particles,^27^ which partially elucidated the anticoagulant capacity of XLHDL particles. These observations are further supported by existing mechanistic evidence. Mineo et al^28^ highlighted HDL’s multifaceted antithrombotic mechanisms, including the suppression of thrombin generation. Furthermore, Fernandez et al^27^ demonstrated that fresh HDL2 subfractions, which referred to relatively LHDL, enhance anticoagulant activity of activated protein C and protein S in plasma clotting assays, aligning with our observations regarding larger HDL particles and lower ETP.

Numerous epidemiological studies have consistently demonstrated that elevated levels of coagulation factors, such as FVIII, FIX, FXI, and fibrinogen, are associated with an increased risk of VTE,^29,30^ which makes these factors suitable for intermediate phenotypes for assessing VTE risk. We found that XLHDL and LHDL were negatively associated with FIX and FXI activities, while MHDL and SHDL showed a positive association with the activities of FVIII, FIX, and FXI. However, considering the varying magnitudes of association observed across different HDL particle size categories (XL, L, and M) for FIX, FXI, and FVIII levels, whether these relationships follow a strictly linear trend or exhibit more threshold-like or binary characteristics warrants further investigation. Beyond these factors, XLHDL, LHDL, and MHDL demonstrated a negative association with fibrinogen levels. This aligns with emerging evidence that HDL may attenuate fibrinogen synthesis via anti-inflammatory pathways (eg, suppressing interleukin-6 signaling) or by modulating fibrin polymerization kinetics through phospholipid-mediated interactions.^10,31,32^ However, the coagulation cascade is a complex network involving many additional factors, including FVII, FX, and FII, as well as anticoagulants, which also contribute to global coagulation levels. Specifically, van der Stoep et al^33^ revealed HDL’s regulatory role in the protein C and S pathways, as well as in the tissue factor pathway inhibitor, providing mechanistic insights into how HDL subclasses might modulate the coagulation system. Therefore, incorporating broader panels of coagulation measurements may help further clarify the specific contribution of HDL particle characteristics to hypercoagulability and downstream clinical outcomes.

We observed sex differences in the size-dependent association between HDL components and coagulation parameters. Previous studies suggest that menopause may reduce LHDL levels due to declining estradiol. For example, Vaisar et al^34^ observed that transdermal estradiol supplementation in perimenopausal women increased HDL particle size but reduced cholesterol efflux capacity. Similarly, Beazer et al^35^ reported positive associations between estradiol and larger HDL particles in premenopausal women. However, in our analyses, we adjusted all models for menopausal status but still observed distinct associations in women compared with men. Differences in HDL profiles by menopausal status were subtle, indicating that the sex-specific associations with coagulation likely reflect broader physiological mechanisms beyond menopause.

Several limitations should be acknowledged. First, the cross-sectional design limits causal inference though our findings align with longitudinal data from Lee et al,^8^ complementing their Mendelian randomization and nonfasting lipid results by using directly measured hemostatic parameters in fasting samples. Second, experimental validation of HDL particle size effects on coagulation was not feasible, despite supportive mechanistic evidence. Third, the study population was middle-aged and primarily of European ancestry (45–65 years), limiting generalizability to other ages or ethnicities. Fourth, CRP adjustment may underestimate associations if CRP acts as a mediator rather than a confounder. Fifth, thrombin generation assay standardization remains imperfect, affecting clinical applicability. Last, platelet distribution width is an indirect, nonspecific marker of platelet activation; direct measures like P-selectin would be more informative.

Despite these limitations, the study has notable strengths. It is the first to link HDL characteristics with a broad panel of hemostatic parameters in a large population-based cohort. High-throughput ^1^H-NMR metabolomics allowed detailed HDL quantification, and thrombin generation assays captured both procoagulant and anticoagulant activity, reflecting overall coagulation balance. The large sample size enabled adjustment for multiple confounders, including demographics, lifestyle, and systemic inflammation.

## Conclusions

In conclusion, our data demonstrate size-dependent associations between HDL particles and coagulation, with larger HDL associated with lower levels of procoagulants and reduced thrombin generation potential, whereas medium small and SHDL components showed the opposite pattern. These findings complement previous longitudinal and genetic studies, providing direct mechanistic evidence that strengthens the biological plausibility of HDL characteristics as determinants of thrombosis risk.

## ARTICLE INFORMATION

### Acknowledgments

The authors express their gratitude to all the study participants.

### Sources of Funding

The NEO study (the Netherlands Epidemiology of Obesity) is supported by the participating departments, the Division and the Board of Directors of the Leiden University Medical Center, and by the Leiden University, Research Profile Area Vascular and Regenerative Medicine. Coagulation factor analyses were funded by Stichting de Merel. Stichting de Merel had no role in the study design, data collection, and analysis, decision to publish, or preparation, review, or approval of the manuscript. U.J.F. Tietge is supported by grants from the Swedish Heart-Lung Foundation (20220271 and 20241339) and ALF Medicin by Region Stockholm (project grant FOUI-962738). L. Yuan is sponsored by the Shanghai Pujiang Program (grant 24PJD090).

### Disclosures

H. Vink works for the Glycocalyx Research Institute. The other authors report no conflicts.

### Supplemental Material

Supplemental Materials and Methods

Tables S1–S15

Figures S1–S4

## Supplementary Material
