# Supplementary material for: Association Between High-Density Lipoprotein Characteristics and Hemostatic Parameters in the Netherlands Epidemiology of Obesity (NEO) Study—Brief Report
Source: Arterioscler Thromb Vasc Biol. 2026 Jan 21;46(2):e323515. doi: 10.1161/ATVBAHA.125.323515 (PMC12822762; doi:10.1161/ATVBAHA.125.323515)
Supplement: Supplementary file 1 [file atv-46-e323515-s001.pdf]

SUPPLEMENTAL MATERIALS

The association between high-density lipoprotein characteristics and hemostatic parameters in the Netherlands Epidemiology of Obesity (NEO) Study

Short title: HDL characteristics and hemostatic parameters (45/50)

Lushun Yuan, MD, PhD#[1,2], Jihee Han, MS#[3], Shuzhen Cheng, PhD[4,5], Frits R. Rosendaal, MD, PhD[3], Dennis O. Mook-Kanamori, MD, PhD[3,6], J. Wouter Jukema, MD, PhD[7], Hans Vink, PhD[8], Bernard M. van den Berg, PhD[2], Ton J. Rabelink, MD, PhD[2], Astrid van Hylckama Vlieg, PhD[3], Uwe J. F. Tietge, PhD[9,10], Ko Willems van Dijk, PhD[11,12,13], Ruifang Li-Gao, PhD[3]

1. Department of Vascular Surgery, Intervention Center, Shanghai General Hospital, Shanghai Jiao Tong University School of Medicine, Shanghai, People's Republic of China.

2. Einthoven Laboratory for Vascular and Regenerative Medicine, Department of Internal Medicine, Nephrology, Leiden University Medical Center, Leiden, the Netherlands.

3. Department of Clinical Epidemiology, Leiden University Medical Center, Leiden, the Netherlands.

4. SKL of Marine Food Processing & Safety Control, School of Food Science and Technology, Dalian Polytechnic University, Dalian, Liaoning 116034, People's Republic of China.

5. National Engineering Research Center of Seafood, Collaborative Innovation Center of Seafood Deep Processing, Dalian Polytechnic University, Dalian, Liaoning 116034, People's Republic of China.

6. Department of Public Health and Primary Care, Leiden University Medical Center, Leiden, The Netherlands.

7. Department of Cardiology, Leiden University Medical Center, Leiden, the Netherlands; Netherlands Heart Institute, Utrecht, the Netherlands.

8. Glycocalyx Research Institute, Alpine, Utah, USA.

9. Division of Clinical Chemistry, Department of Laboratory Medicine, Karolinska Institutet, 14183 Stockholm, Sweden.

10. Clinical Chemistry, Karolinska University Laboratory, Karolinska University Hospital, Stockholm, Sweden.

11. Department of Human Genetics, Leiden University Medical Center, Leiden, the Netherlands.

12. Department of Internal Medicine, Division of Endocrinology, Leiden University Medical Center, Leiden, the Netherlands.

[13.] Leiden Laboratory for Experimental Vascular Medicine, Leiden University Medical Center, Leiden, the Netherlands.

#: these authors contributed equally to the work

Supplemental Material

1. Major Resources Table
2. Expanded Materials & Methods
3. Figures S1-S4
4. Tables S1-S15

Major Resources Table

In order to allow validation and replication of experiments, all essential research materials listed in the Methods should be included in the Major Resources Table below. Authors are encouraged to use public repositories for protocols, data, code, and other materials and provide persistent identifiers and/or links to repositories when available. Authors may add or delete rows as needed.

Animals (in vivo studies)

Species	Vendor or Source	Background Strain	Sex	Persistent ID / URL
NA	NA	NA	NA	NA

Genetically Modified Animals

	Species	Vendor or Source	Background Strain	Other Information	Persistent ID / URL
**Parent - Male**	NA	NA	NA	NA	NA
**Parent - Female**	NA	NA	NA	NA	NA

Antibodies

Target antigen	Vendor or Source	Catalog #	Working concentration	Lot # (preferred but not required)	Persistent ID / URL
NA	NA	NA	NA	NA	NA

DNA/cDNA Clones

Clone Name	Sequence	Source / Repository	Persistent ID / URL
NA	NA	NA	NA

Cultured Cells

Name	Vendor or Source	Sex (F, M, or unknown)	Persistent ID / URL
NA	NA	NA	NA

Data & Code Availability

Description	Source / Repository	Persistent ID / URL
NA	NA	NA

Other

Description	Source / Repository	Persistent ID / URL
NA	NA	NA

DOI [to be added]

ARRIVE GUIDELINES

The ARRIVE guidelines (https://arriveguidelines.org/) are a checklist of recommendations to improve the reporting of research involving animals. Key elements of the study design should be included below to better enable readers to scrutinize the research adequately, evaluate its methodological rigor, and reproduce the methods or findings.

Study Design

**Groups**	**Sex**	**Age**	**Number (prior to experiment)**	**Number (after termination)**	**Littermates (Yes/No)**	**Other description**
Group 1 (Control)	NA	NA	NA	NA	NA	NA
Group 2	NA	NA	NA	NA	NA	NA
Add more if needed						

Sample Size: Please explain how the sample size was decided Please provide details of any a *prior* sample size calculation, if done.

NA

Inclusion Criteria

NA

Exclusion Criteria

NA

Randomization

NA

Blinding

NA

DOI [to be added]

Expanded Materials & Methods

Statistical analyses

In the NEO study, there is an oversampling of individuals with a BMI of 27 kg/m^2 or higher. To correctly represent associations in the general population, adjustments for this oversampling were made[1]. This was done by weighing all participants towards the BMI distribution of participants from the Leiderdorp municipality[2], whose BMI distribution was similar to the BMI distribution of the Dutch general population[3]. All analyses were based on weighted analyses, and consequently results apply to the general population without oversampling of high BMI. As a result of the weighted analyses, percentages and proportions are given instead of numbers of participants. Other baseline characteristics are expressed as median with interquartile range.

The distributions of confounding factors and HDL parameters were first evaluated, and a z-transformation (i.e., with mean = 0 and standard deviation, SD = 1) was performed on HDL parameters. On inspection of distributions, the velocity of thrombin generation potential showed a right-skewed distribution and natural-log transformation was performed. We reversed lag time and time-to-peak to obtain a consistent interpretation so that positive effect estimates, i.e., increases in levels, are indicative of a procoagulant effect across all hemostatic parameters.

We used linear regression analyses to evaluate the associations between HDL parameters (exposures) and hemostatic factors (outcomes), including procoagulants, thrombin generation potential parameters, and platelet activation parameters. The analyses were adjusted for several potential confounding factors in a stepwise manner. First, crude analyses were performed (model 1). In model 2, we adjusted for age (continuous, year) and sex (categorical, women and men). Model 3 was additionally adjusted for BMI (continuous, kg/m^2), race (categorical, white and others), menopausal status (categorical, premenopausal and peri or postmenopausal), current use of lipid-lowering drugs (categorical, current users and noncurrent users), total body fat (continuous, %), and serum CRP levels (continuous, mg/L). The regression coefficient (β) and 95% confidence intervals (CIs) represent the difference in hemostatic parameter levels associated with one SD of the HDL parameter. To account for multiple testing, false discovery rate correction was applied to the raw p-values in each model, and associations with false discovery rate corrected q-value < 0.05 were considered statistically significant. Furthermore, the associations between HDL and hemostatic parameters were examined using sex-stratified analyses in model 3.

We hypothesized that the endothelial glycocalyx perturbation might be involved in any observed associations between HDL characteristics and hemostatic factors. We first evaluated the associations between HDL characteristics (exposure) and glycocalyx-related parameters (outcome). For any

observed association between HDL characteristics and a glycocalyx-related parameter, we aimed to conduct formal mediation analyses when the four assumptions of the Baron-Kenny framework were met. All the analyses were performed with R version 4.4.0.

References

1. Korn EL and Graubard BI. Epidemiologic studies utilizing surveys: accounting for the sampling design. *Am J Public Health*. 1991;81:1166-73.
2. Lumley T. Analysis of complex survey samples. *Journal of statistical software*. 2004;9:1-19.
3. Volksgezondheid Mv. Hoeveel mensen hebben overgewicht? (How many people are overweight?), www.rivm.nl/nldemaat [accessed 2013.05.13]. 2013.

Supplemental Figures

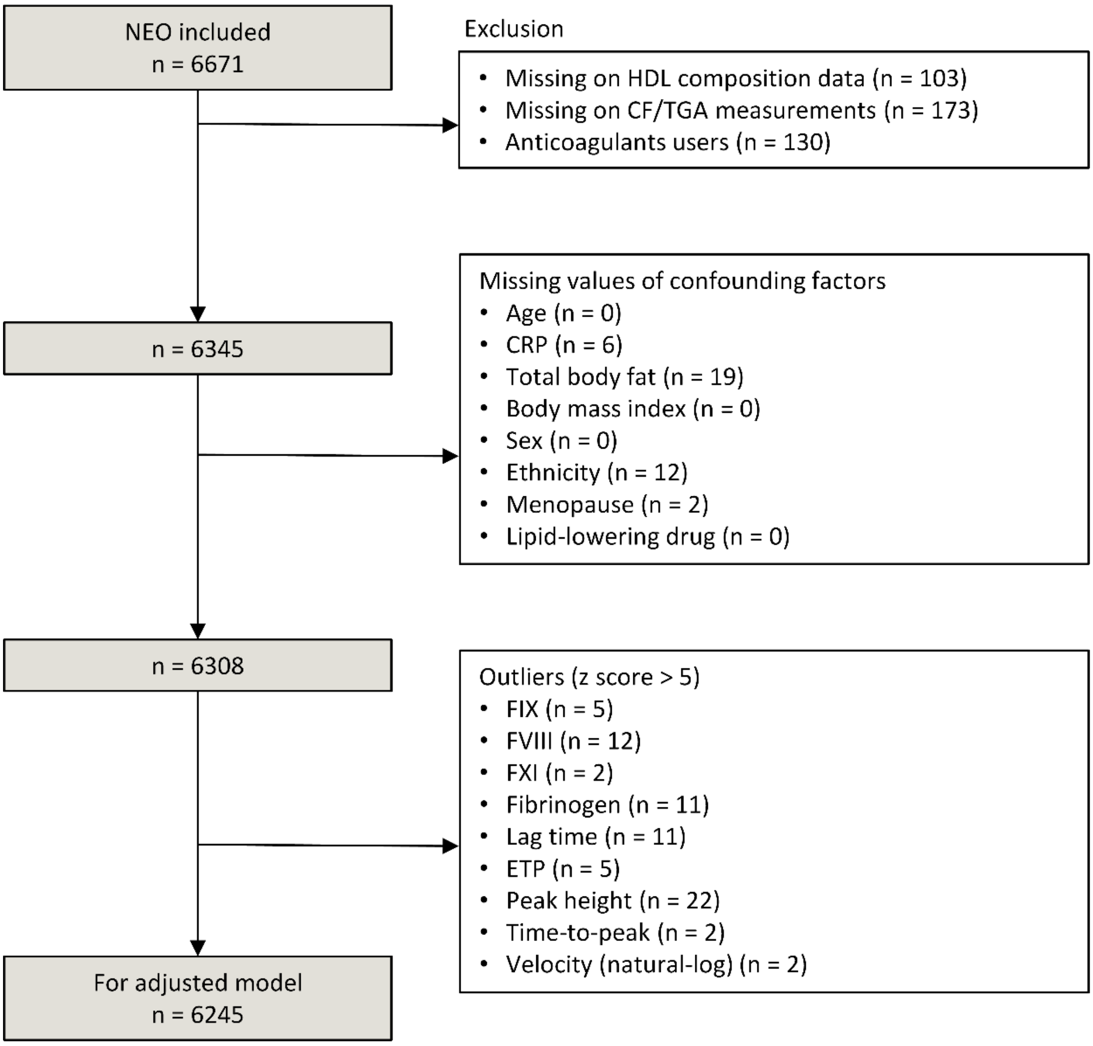

Figure S1. Study workflow for analysing the associations between HDL characteristics and coagulation parameters, with participants included in multiple exclusion criteria at each step

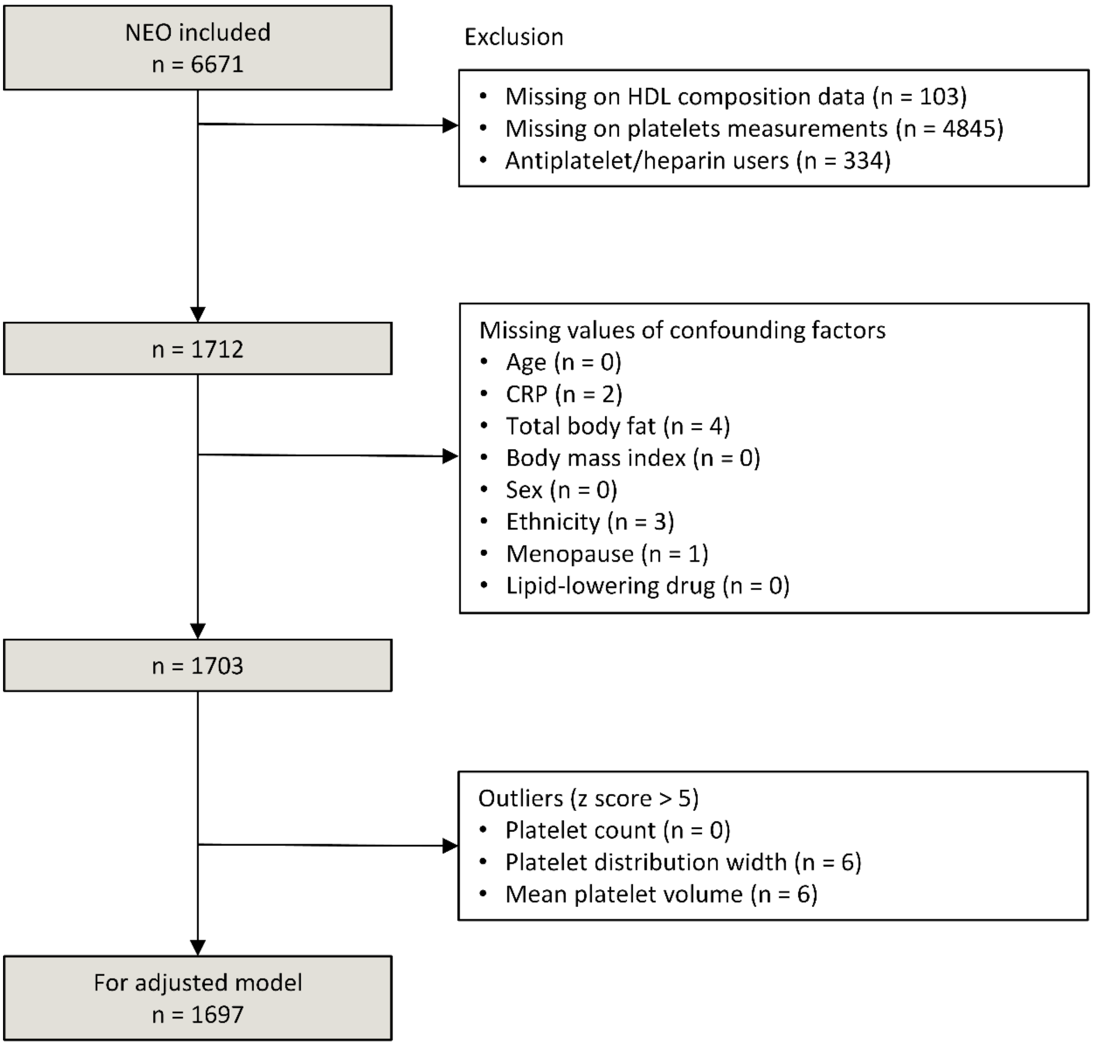

Figure S2. Study workflow for analysing the associations between HDL composition and platelet activation parameters, with participants included in multiple exclusion criteria at each step.

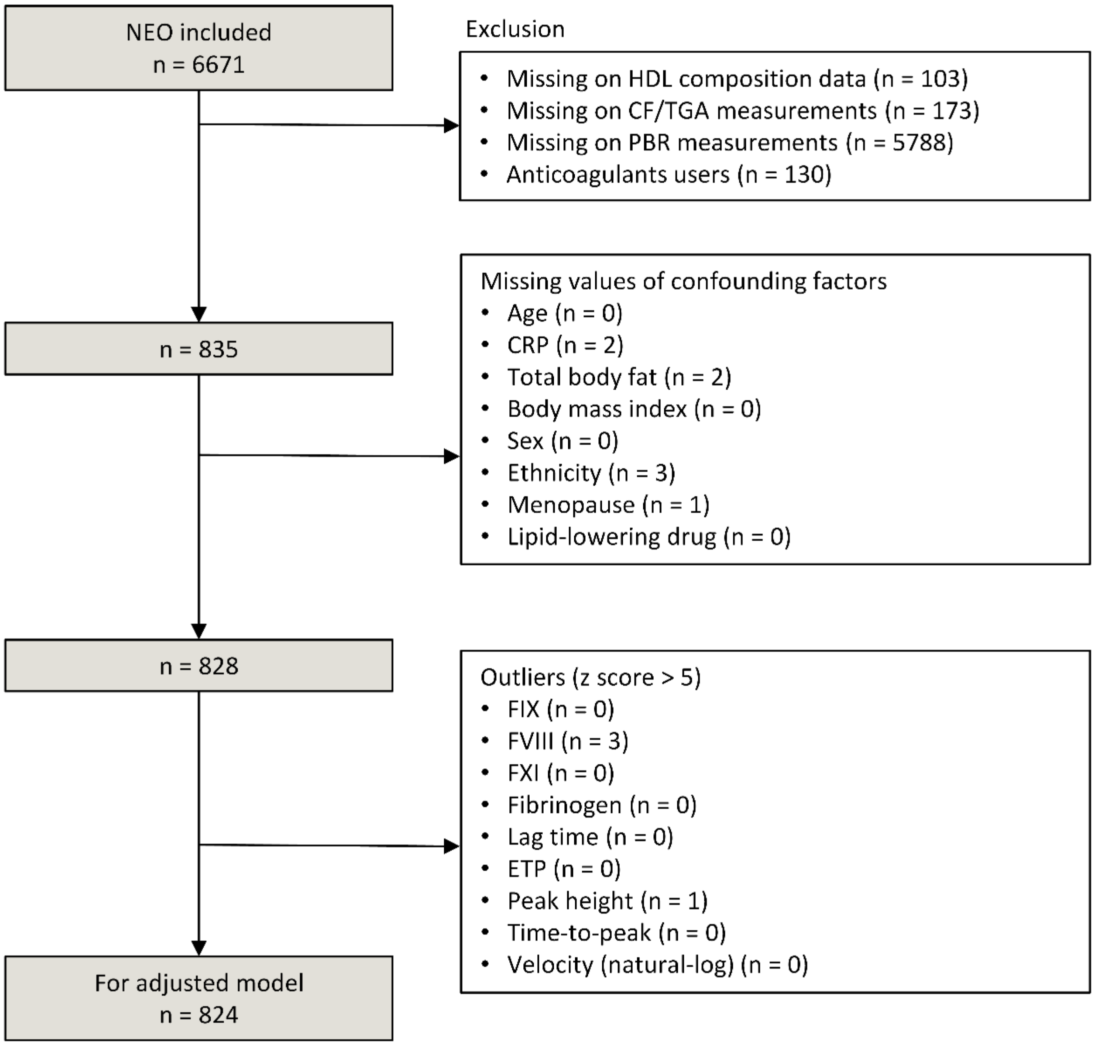

Figure S3. Study workflow for analysing the associations between HDL composition and glycocalyx-related parameters (i.e., PBR), with participants included in multiple exclusion criteria at each step.

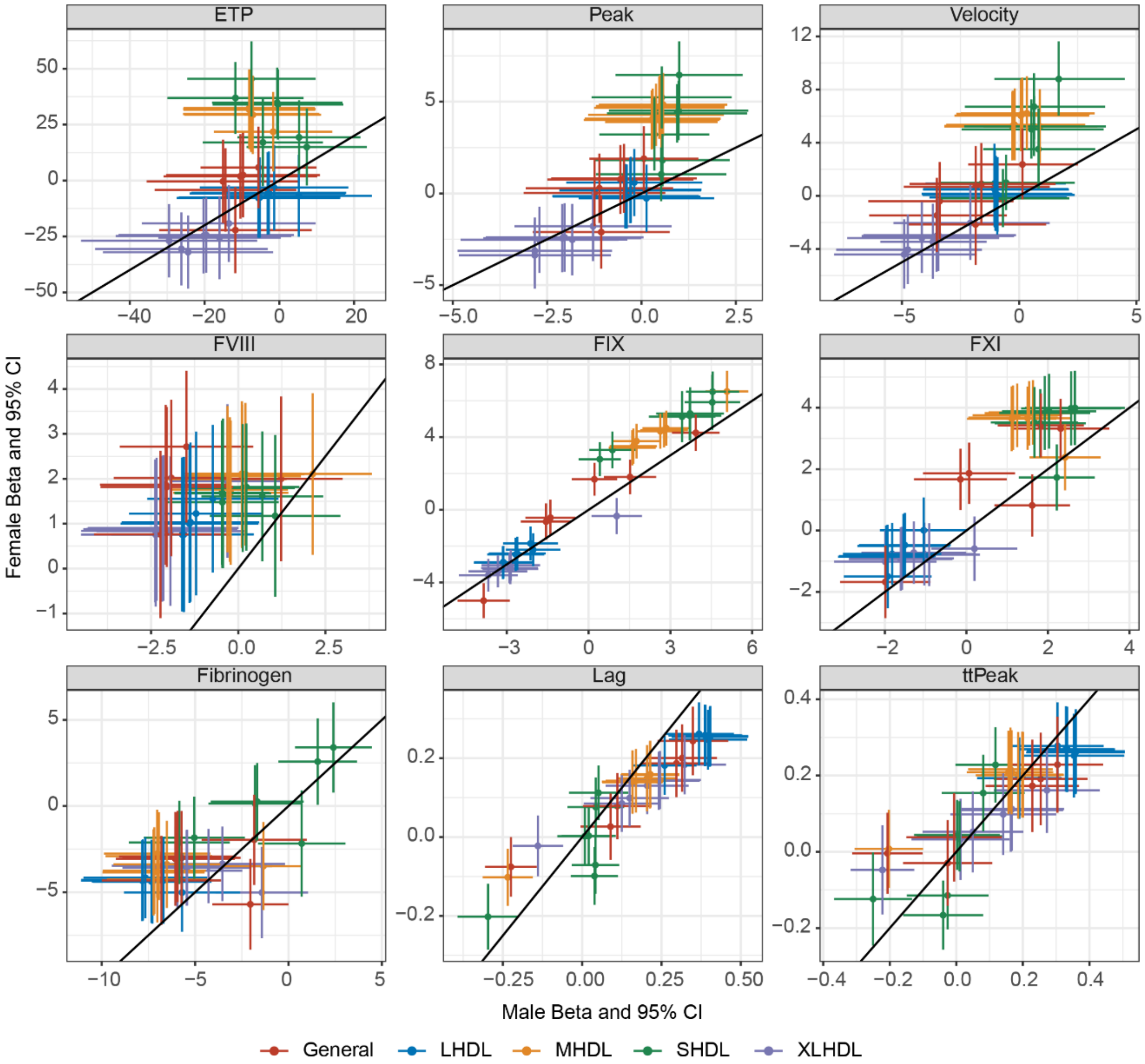

Figure S4. Beta-beta plot comparing the effect estimates of HDL composition associated with coagulation parameters between men and women. Overall, effect estimates of HDL composition were stronger in women than men. The effect size and a 95% confidence interval were depicted by a dot with a horizontal line in men and a dot with a vertical line in women. Different colors represent different HDL compositions. Lag time and time-to-peak were reversed to obtain a consistent interpretation indicating that positive effect estimates, i.e., increases in levels, are indicative of a procoagulant effect across all hemostatic parameters.

Supplemental Tables

Table S1. Detailed abbreviations of HDL composition

Labels	Unit	Abbreviation
**Lipoprotein particle sizes**		
Average diameter for HDL particles	nm	HDLD
**Lipoprotein particle concentrations**		
Total cholesterol in HDL	mmol/l	HDLC
Total cholesterol in HDL2, particle density 1.063	mmol/l	HDL2C
Total cholesterol in HDL3, particle density 1.125	mmol/l	HDL3C
Total triglycerides in HDL	mmol/l	HDLTG
**Apolipoproteins**		
Apolipoprotein A1	g/l	ApoA1
**Very large HDL (average diameter 14.3 nm)**		
Concentration of very large HDL particles	mmol/l	XLHDLP
Total lipids in very large HDL	mmol/l	XLHDLL
Phospholipids in very large HDL	mmol/l	XLHDLPL
Cholesterol in very large HDL	mmol/l	XLHDLC
Cholesteryl esters in very large HDL	mmol/l	XLHDLCE
Free cholesterol in very large HDL	mmol/l	XLHDLFC
Triglycerides in very large HDL	mmol/l	XLHDLTG
**Large HDL (average diameter 12.1 nm)**		
Concentration of large HDL particles	mmol/l	LHDLP
Total lipids in large HDL	mmol/l	LHDLL
Phospholipids in large HDL	mmol/l	LHDLPL
Cholesterol in large HDL	mmol/l	LHDLC
Cholesteryl esters in large HDL	mmol/l	LHDLCE
Free cholesterol in large HDL	mmol/l	LHDLFC
Triglycerides in large HDL	mmol/l	LHDLTG
**Medium HDL (average diameter 10.9 nm)**		
Concentration of medium HDL particles	mmol/l	MHDLP
Total lipids in medium HDL	mmol/l	MHDLL
Phospholipids in medium HDL	mmol/l	MHDLPL
Cholesterol in medium HDL	mmol/l	MHDLC
Cholesteryl esters in medium HDL	mmol/l	MHDLCE
Free cholesterol in medium HDL	mmol/l	MHDLFC
Triglycerides in medium HDL	mmol/l	MHDLTG
**Small HDL (average diameter 8.7 nm)**		
Concentration of small HDL particles	mmol/l	SHDLP
Total lipids in small HDL	mmol/l	SHDLL
Phospholipids in small HDL	mmol/l	SHDLPL
Cholesterol in small HDL	mmol/l	SHDLC
Cholesteryl esters in small HDL	mmol/l	SHDLCE
Free cholesterol in small HDL	mmol/l	SHDLFC
Triglycerides in small HDL	mmol/l	SHDLTG

Table S2. Characteristics of the study population

	Study population (n = 6245)
**Demography**	
Age (year)	57 (51 - 61)
Sex (% women)	56
BMI (kg/m2)	24.6 (22.85 - 26.20)
Total body fat (%)	30.2 (22.9 - 36.5)
Race (% white)	95
Menopause status (% peri and postmenopausal, in women)	81.7
C-Reactive protein (mg/L)	0.95 (0.50 - 1.86)
Use of lipid-lowering drugs (% current use)	10.2
**The levels of endothelial glycocalyx**	
PBR capillaries (µm, n = 851)	1.21 (1.14 - 1.26)
PBR feed vessels (µm, n = 851)	2.30 (2.11 - 2.49)
PBR total (µm, n = 812)	2.34 (2.20 - 2.51)
**Platelet activation parameters**	
Platelet count (billion/L, n = 1757)	240 (204 - 273)
Platelet distribution width (%, n = 1696)	12.2 (11.3 - 13.3)
Mean platelet volume (fL, n = 1696)	10.5 (10.0 - 11.0)
**Coagulation factors**	
FVIII activity (%)	121.90 (103.17 - 144.39)
FIX activity (%)	114.32 (102.83 - 125.88)
FXI activity (%)	113.62 (100.97 - 125.65)
Fibrinogen (mg/dL)	275.71 (244.70 - 308.59)
**The parameters of thrombin generation potential**	
Endogenous thrombin potential (nM·min)	1108 (885 - 1317)
Peak height (nM)	80.13 (59.91 - 100.44)
Lag time (min)	6.50 (5.75 - 7.48)
Time to peak (min)	14.50 (13.31 - 15.74)
Velocity (nM/min)	10.07 (7.13 - 13.73)

Because of the weighted analyses, percentages and proportions are given instead of numbers of participants. Other baseline characteristics are expressed as median with interquartile range and interquartile range.

Table S3. Characteristics of the study population that were used to examine the associations between HDL composition and platelet activation parameters.

	Study population (n = 1697)
**Demography**	
Age (year)	57 (51 - 62)
Sex (% women)	57.8
BMI (kg/m2)	24.60 (22.84 - 26.25)
Total body fat (%)	30.20 (22.50 - 36.10)
Race (% white)	95.6
Menopause status (% peri and postmenopausal, in women)	81.5
C-Reactive protein (mg/L)	0.93 (0.50 - 1.88)
Use of lipid-lowering drugs (% current use)	5.3
**The levels of endothelial glycocalyx**	
PBR capillaries (µm, n = 851)	1.20 (1.14 - 1.26)
PBR feed vessels (µm, n = 851)	2.30 (2.10 - 2.48)
PBR total (µm, n = 812)	2.34 (2.20 - 2.51)
**Platelet activation parameters**	
Platelet count (billion/L, n = 1757)	240 (204 - 273)
Platelet distribution width (%, n = 1696)	12.2 (11.3 - 13.3)
Mean platelet volume (fL, n = 1696)	10.5 (10.0 - 11.1)
**Coagulation factors**	
FVIII activity (%)	122.98 (104.07 - 145.69)
FIX activity (%)	114.32 (101.75 - 125.88)
FXI activity (%)	113.62 (100.97 - 125.65)
Fibrinogen (mg/dL)	275.71 (244.70 - 308.59)
**The parameters of thrombin generation potential**	
Endogenous thrombin potential (nM·min)	1103.70 (877.45 - 1313.55)
Peak height (nM)	79.61 (58.90 - 100.44)
Lag time (min)	6.50 (5.75 - 7.50)
Time to peak (min)	14.50 (13.33 - 15.75)
Velocity (nM/min)	10.04 (6.99 - 13.72)

Because of the weighted analyses, percentages and proportions are given instead of numbers of participants. Other baseline characteristics are expressed as median with interquartile range and interquartile range.

Table S4. Characteristics of the study population that were used to examine the associations between HDL composition and glycocalyx-related parameters.

	Study population (n = 824)
**Demography**	
Age (year)	57 (51 - 61)
Sex (% women)	55.5
BMI (kg/m2)	24.63 (22.85 - 26.20)
Total body fat (%)	30.20 (22.90 - 36.20)
Race (% white)	94.2
Menopause status (% peri and postmenopausal, in women)	80.6
C-Reactive protein (mg/L)	0.95 (0.50 - 1.86)
Use of lipid-lowering drugs (% current use)	7.1
**The levels of endothelial glycocalyx**	
PBR capillaries (µm, n = 851)	1.21 (1.14 - 1.26)
PBR feed vessels (µm, n = 851)	2.30 (2.11 - 2.49)
PBR total (µm, n = 812)	2.34 (2.20 - 2.51)
**Platelet activation parameters**	
Platelet count (billion/L, n = 1757)	240 (204 - 273)
Platelet distribution width (%, n = 1696)	12.2 (11.3 - 13.3)
Mean platelet volume (fL, n = 1696)	10.5 (10.0 - 11.0)
**Coagulation factors**	
FVIII activity (%)	121.90 (103.17 - 144.39)
FIX activity (%)	114.32 (102.83 - 125.88)
FXI activity (%)	113.62 (100.97 - 125.65)
Fibrinogen (mg/dL)	275.71 (244.70 - 308.59)
**The parameters of thrombin generation potential**	
Endogenous thrombin potential (nM·min)	1108 (885 - 1317)
Peak height (nM)	80.13 (59.91 - 100.44)
Lag time (min)	6.50 (5.75 - 7.48)
Time to peak (min)	14.50 (13.31 - 15.74)
Velocity (nM/min)	10.07 (7.13 - 13.73)

Because of the weighted analyses, percentages and proportions are given instead of numbers of participants. Other baseline characteristics are expressed as median with interquartile range and interquartile range.

Table S5. Associations between HDL composition and levels of coagulation parameters including ETP, peak, velocity, lag time and time to peak, FVIII, FIX, FXI, and fibrinogen in the total population (model 1).

HDL	Abbreviation	ETP	Peak height	Velocity	Lag time	Time to peak	FVIII	FIX	FXI	Fibrinogen
**Lipoprotein particle sizes**										
Average diameter for HDL particles	HDLD	-23.09 (-34.63 - -11.55)	-2.26 (-3.35 - -1.18	-3.00 (-4.70 - -1.27	-0.41 (-0.46 - -0.35	0.41 (0.35 - 0.46)*	-0.88 (-1.98 - 0.2	-6.22 (-6.81 - -5.63	-0.22 (-0.89 - 0.4	-6.17 (-8.11 - -4.23)*
**Lipoprotein particle concentrations**										
Total cholesterol in HDL	HDLC	-15.33 (-27.66 - -2.99)*	-1.17 (-2.35 - 0.02)	-2.12 (-3.95 - -0.25	-0.36 (-0.41 - -0.30	0.36 (0.30 - 0.41)*	-0.04 (-1.19 - 1.1	-3.41 (-4.05 - -2.76	2.04 (1.37 - 2.71	-4.85 (-6.84 - -2.85)*
Total cholesterol in HDL2	HDL2C	-14.59 (-26.86 - -2.32)*	-1.09 (-2.26 - 0.09)	-1.92 (-3.75 - -0.06	-0.37 (-0.43 - -0.31	0.37 (0.31 - 0.43)*	-0.08 (-1.22 - 1.0	-3.58 (-4.23 - -2.94	1.87 (1.20 - 2.54	-5.00 (-7.00 - -3.01)*
Total cholesterol in HDL3	HDL3C	-21.10 (-34.35 - -7.86)*	-1.87 (-3.14 - -0.60	-3.90 (-5.81 - -1.96	-0.17 (-0.23 - -0.11	0.17 (0.11 - 0.23)*	0.30 (-0.88 - 1.4[illegible]	-1.09 (-1.75 - -0.43	3.54 (2.80 - 4.28	-2.56 (-4.55 - -0.57)*
Total triglycerides in HDL	HDLTG	10.63 (-2.36 - 23.62)	1.78 (0.55 - 3.00)*	2.13 (0.29 - 3.99)*	0.21 (0.15 - 0.26)*	-0.21 (-0.26 - -0.15	2.51 (1.20 - 3.83	5.51 (4.80 - 6.22)*	2.32 (1.60 - 3.04	1.30 (-0.74 - 3.33)
**Apolipoproteins**										
Apolipoprotein A1	ApoA1	-17.88 (-30.35 - -5.40)*	-1.63 (-2.83 - -0.42	-3.21 (-5.04 - -1.34	-0.22 (-0.27 - -0.16	0.22 (0.16 - 0.27)*	0.72 (-0.45 - 1.8[illegible]	-1.00 (-1.66 - -0.34	3.66 (2.98 - 4.35	-3.95 (-5.95 - -1.95)*
**Very large HDL (average diameter 14.3 nm)**										
Concentration of very large HDL particle[illegible]	XLHDLP	-27.50 (-38.99 - -16.01)	-2.78 (-3.92 - -1.64	-3.85 (-5.59 - -2.08	-0.31 (-0.36 - -0.25	0.31 (0.25 - 0.36)*	-0.52 (-1.63 - 0.5	-5.23 (-5.84 - -4.62	0.16 (-0.51 - 0.8[illegible]	-5.07 (-6.90 - -3.23)*
Total lipids in very large HDL	XLHDLL	-27.95 (-39.42 - -16.48)	-2.82 (-3.96 - -1.68	-3.90 (-5.64 - -2.14	-0.30 (-0.36 - -0.25	0.30 (0.25 - 0.36)*	-0.54 (-1.65 - 0.5	-5.22 (-5.83 - -4.61	0.17 (-0.50 - 0.8[illegible]	-5.08 (-6.92 - -3.24)*
Phospholipids in very large HDL	XLHDLPL	-22.94 (-34.50 - -11.37)	-2.24 (-3.39 - -1.10	-3.03 (-4.80 - -1.23	-0.35 (-0.40 - -0.30	0.35 (0.30 - 0.40)*	-0.48 (-1.59 - 0.6	-5.40 (-6.01 - -4.79	0.09 (-0.58 - 0.7[illegible]	-5.05 (-6.90 - -3.19)*
Cholesterol in very large HDL	XLHDLC	-33.70 (-45.12 - -22.28)	-3.47 (-4.60 - -2.34	-4.86 (-6.56 - -3.13	-0.25 (-0.30 - -0.20	0.25 (0.20 - 0.30)*	-0.68 (-1.79 - 0.4	-5.07 (-5.67 - -4.47	0.24 (-0.43 - 0.9[illegible]	-5.08 (-6.91 - -3.24)*
Cholesteryl esters in very large HDL	XLHDLCE	-34.89 (-46.40 - -23.37)	-3.68 (-4.82 - -2.54	-5.18 (-6.88 - -3.45	-0.24 (-0.29 - -0.18	0.24 (0.18 - 0.29)*	-0.71 (-1.82 - 0.3	-5.13 (-5.73 - -4.52	0.19 (-0.48 - 0.8[illegible]	-5.01 (-6.85 - -3.18)*
Free cholesterol in very large HDL	XLHDLFC	-30.64 (-41.92 - -19.36)	-2.96 (-4.08 - -1.83	-4.07 (-5.79 - -2.31	-0.29 (-0.34 - -0.23	0.29 (0.23 - 0.34)*	-0.60 (-1.71 - 0.5	-4.89 (-5.49 - -4.30	0.35 (-0.32 - 1.0[illegible]	-5.17 (-7.01 - -3.34)*
Triglycerides in very large HDL	XLHDLTG	-22.19 (-35.24 - -9.13)*	-2.46 (-3.71 - -1.21	-4.02 (-5.84 - -2.17	0.01 (-0.05 - 0.07)	-0.01 (-0.07 - 0.05)	0.68 (-0.51 - 1.8[illegible]	-0.83 (-1.59 - -0.08	0.55 (-0.20 - 1.3[illegible]	-2.69 (-4.79 - -0.59)*
**Large HDL (average diameter 12.1 nm)**										
Concentration of large HDL particles	LHDLP	-13.18 (-25.28 - -1.08)*	-1.00 (-2.16 - 0.16)	-1.52 (-3.34 - 0.33)	-0.41 (-0.47 - -0.36	0.41 (0.36 - 0.47)*	-0.20 (-1.33 - 0.9	-4.59 (-5.23 - -3.95	0.50 (-0.16 - 1.1[illegible]	-5.66 (-7.56 - -3.75)*
Total lipids in large HDL	LHDLL	-13.48 (-25.56 - -1.40)*	-1.02 (-2.18 - 0.14)	-1.54 (-3.36 - 0.31)	-0.41 (-0.47 - -0.36	0.41 (0.36 - 0.47)*	-0.23 (-1.36 - 0.9	-4.63 (-5.27 - -4.00	0.47 (-0.19 - 1.1[illegible]	-5.69 (-7.60 - -3.79)*
Phospholipids in large HDL	LHDLPL	-11.71 (-24.06 - 0.63)	-0.81 (-2.00 - 0.37)	-1.37 (-3.22 - 0.51)	-0.41 (-0.47 - -0.36	0.41 (0.36 - 0.47)*	-0.03 (-1.18 - 1.1	-4.16 (-4.81 - -3.51	0.89 (0.22 - 1.56	-5.36 (-7.29 - -3.43)*
Cholesterol in large HDL	LHDLC	-15.17 (-27.04 - -3.31)*	-1.21 (-2.35 - -0.06	-1.67 (-3.46 - 0.15)	-0.41 (-0.47 - -0.36	0.41 (0.36 - 0.47)*	-0.43 (-1.54 - 0.6	-5.00 (-5.62 - -4.37	0.16 (-0.50 - 0.8[illegible]	-5.95 (-7.84 - -4.07)*
Cholesteryl esters in large HDL	LHDLCE	-14.92 (-26.79 - -3.05)*	-1.19 (-2.33 - -0.05	-1.65 (-3.44 - 0.18)	-0.42 (-0.47 - -0.36	0.42 (0.36 - 0.47)*	-0.42 (-1.54 - 0.6	-5.00 (-5.62 - -4.37	0.14 (-0.52 - 0.8[illegible]	-5.96 (-7.84 - -4.08)*
Free cholesterol in large HDL	LHDLFC	-15.96 (-27.81 - -4.11)*	-1.26 (-2.40 - -0.12	-1.75 (-3.54 - 0.08)	-0.41 (-0.46 - -0.35	0.41 (0.35 - 0.46)*	-0.44 (-1.55 - 0.6	-5.00 (-5.62 - -4.38	0.21 (-0.45 - 0.8[illegible]	-5.93 (-7.81 - -4.06)*
Triglycerides in large HDL	LHDLTG	-6.79 (-18.98 - 5.41)	-0.67 (-1.84 - 0.49)	-1.27 (-3.09 - 0.58)	-0.33 (-0.38 - -0.28	0.33 (0.28 - 0.38)*	0.30 (-0.80 - 1.4[illegible]	-4.05 (-4.72 - -3.38	-0.17 (-0.85 - 0.5	-4.73 (-6.64 - -2.81)*
**Medium HDL (average diameter 10.9 nm)**										
Concentration of medium HDL particles	MHDLP	10.60 (-2.34 - 23.54)	2.19 (0.93 - 3.46)*	2.43 (0.51 - 4.39)*	-0.24 (-0.30 - -0.18	0.24 (0.18 - 0.30)*	1.69 (0.58 - 2.81	2.35 (1.67 - 3.03)*	4.15 (3.38 - 4.91	-3.12 (-5.14 - -1.10)*
Total lipids in medium HDL	MHDLL	9.71 (-3.24 - 22.66)	2.07 (0.81 - 3.33)*	2.26 (0.35 - 4.21)*	-0.25 (-0.31 - -0.19	0.25 (0.19 - 0.31)*	1.59 (0.47 - 2.70	2.08 (1.40 - 2.76)*	4.06 (3.29 - 4.82	-3.26 (-5.28 - -1.24)*
Phospholipids in medium HDL	MHDLPL	10.68 (-2.37 - 23.73)	2.28 (1.00 - 3.57)*	2.55 (0.60 - 4.54)*	-0.24 (-0.30 - -0.18	0.24 (0.18 - 0.30)*	1.70 (0.57 - 2.83	2.23 (1.56 - 2.90)*	4.03 (3.27 - 4.79	-2.99 (-5.00 - -0.98)*
Cholesterol in medium HDL	MHDLC	5.65 (-7.27 - 18.56)	1.44 (0.21 - 2.68)*	1.43 (-0.43 - 3.33)	-0.29 (-0.35 - -0.23	0.29 (0.23 - 0.35)*	1.06 (-0.05 - 2.1[illegible]	0.91 (0.24 - 1.59)*	3.60 (2.84 - 4.37	-3.91 (-5.95 - -1.87)*
Cholesteryl esters in medium HDL	MHDLCE	5.57 (-7.30 - 18.44)	1.41 (0.18 - 2.63)*	1.42 (-0.43 - 3.30)	-0.29 (-0.35 - -0.23	0.29 (0.23 - 0.35)*	1.01 (-0.09 - 2.1[illegible]	0.87 (0.19 - 1.54)*	3.54 (2.78 - 4.31	-4.00 (-6.04 - -1.96)*
Free cholesterol in medium HDL	MHDLFC	5.83 (-7.26 - 18.91)	1.56 (0.30 - 2.82)*	1.47 (-0.44 - 3.41)	-0.28 (-0.34 - -0.22	0.28 (0.22 - 0.34)*	1.22 (0.10 - 2.35	1.08 (0.40 - 1.75)*	3.78 (3.03 - 4.53	-3.54 (-5.57 - -1.51)*
Triglycerides in medium HDL	MHDLTG	24.94 (11.94 - 37.94)*	3.37 (2.13 - 4.61)*	4.46 (2.58 - 6.37)*	0.27 (0.22 - 0.33)*	-0.27 (-0.33 - -0.22	3.25 (2.01 - 4.48	8.10 (7.34 - 8.87)*	3.29 (2.59 - 4.00	3.78 (1.80 - 5.76)*
**Small HDL (average diameter 8.7 nm)**										
Concentration of small HDL particles	SHDLP	25.20 (12.01 - 38.38)*	3.45 (2.18 - 4.73)*	3.82 (1.99 - 5.69)*	0.03 (-0.03 - 0.09)	-0.03 (-0.09 - 0.03)	2.12 (1.06 - 3.17	5.78 (4.52 - 7.04)*	4.33 (3.33 - 5.33	2.31 (0.37 - 4.24)*
Total lipids in small HDL	SHDLL	23.61 (10.38 - 36.84)*	3.24 (1.97 - 4.51)*	3.51 (1.68 - 5.37)*	0.02 (-0.04 - 0.07)	-0.02 (-0.07 - 0.04)	2.00 (0.94 - 3.06	5.42 (4.20 - 6.64)*	4.28 (3.27 - 5.28	2.11 (0.17 - 4.04)*
Phospholipids in small HDL	SHDLPL	37.37 (24.36 - 50.38)*	5.40 (4.07 - 6.72)*	7.38 (5.38 - 9.42)*	-0.07 (-0.13 - -0.01	0.07 (0.01 - 0.13)*	2.42 (1.37 - 3.47	6.73 (5.59 - 7.87)*	3.77 (2.87 - 4.67	0.96 (-0.99 - 2.91)
Cholesterol in small HDL	SHDLC	4.14 (-9.35 - 17.63)	0.34 (-0.88 - 1.57)	-0.88 (-2.68 - 0.95)	0.02 (-0.03 - 0.08)	-0.02 (-0.08 - 0.03)	0.92 (-0.19 - 2.0[illegible]	2.00 (1.20 - 2.80)*	3.55 (2.60 - 4.49	1.82 (-0.13 - 3.76)
Cholesteryl esters in small HDL	SHDLCE	0.26 (-13.25 - 13.78)	-0.34 (-1.57 - 0.90)	-1.87 (-3.69 - -0.01	0.03 (-0.02 - 0.09)	-0.03 (-0.09 - 0.02)	0.73 (-0.40 - 1.8[illegible]	1.25 (0.52 - 1.97)*	3.30 (2.40 - 4.21	2.06 (0.11 - 4.02)
Free cholesterol in small HDL	SHDLFC	23.98 (10.71 - 37.26)*	3.94 (2.62 - 5.25)*	5.01 (3.08 - 6.97)*	-0.06 (-0.12 - -0.00	0.06 (0.00 - 0.12)*	1.67 (0.60 - 2.73	5.43 (4.32 - 6.54)*	3.73 (2.83 - 4.64	-0.11 (-2.02 - 1.80)
Triglycerides in small HDL	SHDLTG	21.36 (9.07 - 33.66)*	2.96 (1.84 - 4.08)*	3.83 (2.08 - 5.61)*	0.40 (0.34 - 0.46)*	-0.40 (-0.46 - -0.34	2.16 (0.91 - 3.41	8.06 (7.24 - 8.88)*	1.97 (1.28 - 2.65	5.25 (3.15 - 7.36)*

In the crude model, differences in the total population between HDL composition and levels of coagulation parameters were observed. The effect size and 95% confidence intervals are presented. After multiple testing correction, significant associations are highlighted with an asterisk (*).

Table S6. Associations between HDL composition and levels of coagulation parameters including ETP, peak, velocity, lag time and time to peak, FVIII, FIX, FXI, and fibrinogen in the total population (model 2).

HDL	Abbreviation	ETP	Peak height	Velocity	Lag time	Time to peak	FVIII	FIX	FXI	Fibrinogen
**Lipoprotein particle sizes**										
Average diameter for HDL particles	HDLD	-44.06 (-57.58 - -30.5	-3.73 (-5.00 - -2.4	-4.31 (-6.25 - -2.3	0.36 (0.30 - 0.42)*	0.35 (0.26 - 0.43)*	-2.09 (-3.33 - -0.8	-7.65 (-8.36 - -6.9	-3.32 (-4.09 - -2.5	-12.53 (-14.76 - -10.29)*
**Lipoprotein particle concentrations**										
Total cholesterol in HDL	HDLC	-30.33 (-44.34 - -16.3	-2.00 (-3.34 - -0.6	-2.87 (-4.94 - -0.7	0.31 (0.24 - 0.37)*	0.28 (0.19 - 0.37)*	-1.20 (-2.47 - 0.08	-4.00 (-4.76 - -3.2	-0.38 (-1.13 - 0.37	-10.98 (-13.26 - -8.71)*
Total cholesterol in HDL2	HDL2C	-29.85 (-43.84 - -15.8	-1.93 (-3.27 - -0.5	-2.65 (-4.73 - -0.5	0.32 (0.26 - 0.39)*	0.30 (0.21 - 0.39)*	-1.22 (-2.50 - 0.05	-4.23 (-4.98 - -3.4	-0.61 (-1.36 - 0.14	-11.20 (-13.48 - -8.93)*
Total cholesterol in HDL3	HDL3C	-29.41 (-43.58 - -15.2	-2.30 (-3.67 - -0.9	-4.43 (-6.47 - -2.3	0.10 (0.04 - 0.16)*	0.01 (-0.07 - 0.10)	-0.74 (-1.98 - 0.50	-1.13 (-1.84 - -0.4	1.87 (1.12 - 2.63)*	-6.90 (-9.08 - -4.72)*
Total triglycerides in HDL	HDLTG	12.23 (-0.46 - 24.93)	1.96 (0.76 - 3.17)*	2.36 (0.54 - 4.21)*	-0.21 (-0.26 - -0.1	-0.17 (-0.24 - -0.1	2.18 (0.90 - 3.46)*	5.47 (4.76 - 6.18)*	1.93 (1.23 - 2.63)*	0.38 (-1.72 - 2.49)
**Apolipoproteins**										
Apolipoprotein A1	ApoA1	-30.46 (-44.50 - -16.4	-2.36 (-3.74 - -0.9	-4.02 (-6.08 - -1.9	0.14 (0.08 - 0.21)*	0.07 (-0.02 - 0.16)	-0.27 (-1.55 - 1.00	-0.96 (-1.70 - -0.2	1.81 (1.07 - 2.56)*	-9.53 (-11.78 - -7.28)*
**Very large HDL (average diameter 14.3 nm)**										
Concentration of very large HDL particle	XLHDLP	-45.71 (-58.93 - -32.4	-4.09 (-5.43 - -2.7	-5.11 (-7.07 - -3.1	0.24 (0.18 - 0.30)*	0.21 (0.12 - 0.29)*	-1.55 (-2.78 - -0.3	-6.22 (-6.94 - -5.5	-2.42 (-3.18 - -1.6	-10.36 (-12.45 - -8.26)*
Total lipids in very large HDL	XLHDLL	-46.22 (-59.42 - -33.0	-4.14 (-5.47 - -2.8	-5.17 (-7.13 - -3.1	0.24 (0.18 - 0.30)*	0.20 (0.12 - 0.29)*	-1.57 (-2.80 - -0.3	-6.21 (-6.93 - -5.4	-2.40 (-3.16 - -1.6	-10.36 (-12.45 - -8.26)*
Phospholipids in very large HDL	XLHDLPL	-41.24 (-54.64 - -27.8	-3.51 (-4.86 - -2.1	-4.18 (-6.18 - -2.1	0.30 (0.24 - 0.36)*	0.28 (0.19 - 0.36)*	-1.55 (-2.79 - -0.3	-6.53 (-7.27 - -5.7	-2.68 (-3.44 - -1.9	-10.68 (-12.81 - -8.55)*
Cholesterol in very large HDL	XLHDLC	-50.95 (-63.88 - -38.0	-4.76 (-6.07 - -3.4	-6.15 (-8.03 - -4.2	0.18 (0.12 - 0.24)*	0.12 (0.04 - 0.21)*	-1.64 (-2.85 - -0.4	-5.86 (-6.55 - -5.1	-2.06 (-2.81 - -1.3	-9.78 (-11.82 - -7.73)*
Cholesteryl esters in very large HDL	XLHDLCE	-51.14 (-64.03 - -38.2	-4.91 (-6.22 - -3.6	-6.42 (-8.28 - -4.5	0.16 (0.10 - 0.22)*	0.10 (0.02 - 0.18)*	-1.63 (-2.82 - -0.4	-5.84 (-6.53 - -5.1	-2.01 (-2.75 - -1.2	-9.42 (-11.44 - -7.39)*
Free cholesterol in very large HDL	XLHDLFC	-49.71 (-62.73 - -36.6	-4.32 (-5.64 - -3.0	-5.39 (-7.33 - -3.4	0.22 (0.16 - 0.28)*	0.19 (0.10 - 0.27)*	-1.65 (-2.88 - -0.4	-5.81 (-6.52 - -5.1	-2.16 (-2.92 - -1.4	-10.49 (-12.56 - -8.41)*
Triglycerides in very large HDL	XLHDLTG	-27.03 (-40.18 - -13.8	-2.75 (-4.03 - -1.4	-4.25 (-6.09 - -2.3	-0.07 (-0.12 - -0.0	-0.13 (-0.21 - -0.0	0.30 (-0.87 - 1.48)	-0.71 (-1.47 - 0.04	-0.53 (-1.27 - 0.21	-4.51 (-6.66 - -2.36)*
**Large HDL (average diameter 12.1 nm)**										
Concentration of large HDL particles	LHDLP	-29.13 (-43.04 - -15.2	-1.91 (-3.23 - -0.6	-2.24 (-4.28 - -0.1	0.38 (0.32 - 0.44)*	0.38 (0.30 - 0.46)*	-1.27 (-2.54 - 0.01	-5.50 (-6.27 - -4.7	-2.31 (-3.06 - -1.5	-11.82 (-14.02 - -9.61)*
Total lipids in large HDL	LHDLL	-29.53 (-43.41 - -15.6	-1.95 (-3.26 - -0.6	-2.27 (-4.30 - -0.1	0.38 (0.32 - 0.44)*	0.38 (0.30 - 0.46)*	-1.29 (-2.57 - -0.0	-5.56 (-6.32 - -4.8	-2.34 (-3.09 - -1.5	-11.84 (-14.04 - -9.64)*
Phospholipids in large HDL	LHDLPL	-26.74 (-40.83 - -12.6	-1.63 (-2.95 - -0.3	-2.00 (-4.06 - 0.10	0.38 (0.31 - 0.44)*	0.38 (0.29 - 0.46)*	-1.11 (-2.39 - 0.17	-4.96 (-5.73 - -4.2	-1.86 (-2.62 - -1.1	-11.57 (-13.80 - -9.35)*
Cholesterol in large HDL	LHDLC	-31.85 (-45.51 - -18.1	-2.21 (-3.50 - -0.9	-2.47 (-4.48 - -0.4	0.38 (0.32 - 0.44)*	0.38 (0.30 - 0.46)*	-1.47 (-2.73 - -0.2	-5.99 (-6.74 - -5.2	-2.65 (-3.39 - -1.9	-11.93 (-14.10 - -9.76)*
Cholesteryl esters in large HDL	LHDLCE	-31.58 (-45.26 - -17.9	-2.19 (-3.49 - -0.9	-2.44 (-4.45 - -0.3	0.38 (0.32 - 0.44)*	0.38 (0.30 - 0.47)*	-1.47 (-2.72 - -0.2	-5.99 (-6.74 - -5.2	-2.67 (-3.42 - -1.9	-11.94 (-14.11 - -9.76)*
Free cholesterol in large HDL	LHDLFC	-32.64 (-46.25 - -19.0	-2.26 (-3.55 - -0.9	-2.55 (-4.56 - -0.5	0.37 (0.31 - 0.43)*	0.37 (0.29 - 0.46)*	-1.49 (-2.75 - -0.2	-6.00 (-6.74 - -5.2	-2.57 (-3.31 - -1.8	-11.89 (-14.05 - -9.74)*
Triglycerides in large HDL	LHDLTG	-19.03 (-32.70 - -5.37	-1.39 (-2.70 - -0.0	-1.83 (-3.83 - 0.21	0.27 (0.22 - 0.33)*	0.27 (0.19 - 0.35)*	-0.46 (-1.66 - 0.73	-4.60 (-5.38 - -3.8	-2.77 (-3.51 - -2.0	-9.65 (-11.82 - -7.49)*
**Medium HDL (average diameter 10.9 nm)**										
Concentration of medium HDL particles	MHDLP	7.13 (-6.56 - 20.83)	2.42 (1.09 - 3.75)*	2.97 (0.91 - 5.06)*	0.18 (0.12 - 0.24)*	0.23 (0.14 - 0.31)*	0.95 (-0.24 - 2.14)	2.95 (2.21 - 3.70)*	2.57 (1.78 - 3.35)*	-7.48 (-9.62 - -5.33)*
Total lipids in medium HDL	MHDLL	5.92 (-7.82 - 19.66)	2.27 (0.94 - 3.60)*	2.76 (0.71 - 4.85)*	0.19 (0.13 - 0.25)*	0.24 (0.15 - 0.32)*	0.83 (-0.36 - 2.02)	2.65 (1.91 - 3.40)*	2.46 (1.67 - 3.24)*	-7.65 (-9.81 - -5.50)*
Phospholipids in medium HDL	MHDLPL	7.26 (-6.60 - 21.12)	2.56 (1.21 - 3.92)*	3.16 (1.07 - 5.30)*	0.18 (0.12 - 0.24)*	0.24 (0.15 - 0.32)*	0.93 (-0.28 - 2.13)	2.84 (2.10 - 3.59)*	2.38 (1.61 - 3.16)*	-7.54 (-9.67 - -5.41)*
Cholesterol in medium HDL	MHDLC	0.43 (-13.31 - 14.18)	1.45 (0.15 - 2.75)*	1.70 (-0.31 - 3.74)	0.23 (0.16 - 0.29)*	0.26 (0.17 - 0.34)*	0.30 (-0.88 - 1.49)	1.30 (0.56 - 2.04)*	1.98 (1.20 - 2.76)*	-8.15 (-10.35 - -5.95)*
Cholesteryl esters in medium HDL	MHDLCE	0.31 (-13.37 - 13.98)	1.39 (0.10 - 2.68)*	1.65 (-0.33 - 3.68)	0.23 (0.17 - 0.29)*	0.26 (0.17 - 0.34)*	0.27 (-0.91 - 1.45)	1.24 (0.50 - 1.98)*	1.94 (1.16 - 2.72)*	-8.10 (-10.30 - -5.90)*
Free cholesterol in medium HDL	MHDLFC	0.87 (-13.13 - 14.86)	1.65 (0.31 - 2.99)*	1.82 (-0.24 - 3.92)	0.22 (0.15 - 0.28)*	0.25 (0.16 - 0.34)*	0.40 (-0.81 - 1.61)	1.51 (0.76 - 2.25)*	2.08 (1.30 - 2.86)*	-8.18 (-10.37 - -5.98)*
Triglycerides in medium HDL	MHDLTG	29.68 (16.78 - 42.57)*	3.76 (2.51 - 5.00)*	4.84 (2.95 - 6.76)*	-0.24 (-0.30 - -0.1	-0.18 (-0.25 - -0.1	3.03 (1.81 - 4.26)*	7.94 (7.18 - 8.70)*	3.42 (2.72 - 4.12)*	3.56 (1.51 - 5.61)*
**Small HDL (average diameter 8.7 nm)**										
Concentration of small HDL particles	SHDLP	28.58 (15.41 - 41.74)*	3.80 (2.50 - 5.10)*	4.23 (2.36 - 6.14)*	-0.02 (-0.08 - 0.03	0.00 (-0.07 - 0.08)	1.68 (0.61 - 2.74)*	5.71 (4.45 - 6.96)*	3.94 (3.01 - 4.88)*	1.21 (-0.71 - 3.13)
Total lipids in small HDL	SHDLL	26.75 (13.54 - 39.95)*	3.58 (2.28 - 4.87)*	3.90 (2.04 - 5.80)*	-0.01 (-0.07 - 0.04	0.01 (-0.07 - 0.08)	1.55 (0.49 - 2.62)*	5.35 (4.13 - 6.58)*	3.85 (2.91 - 4.79)*	0.95 (-0.98 - 2.87)
Phospholipids in small HDL	SHDLPL	38.98 (26.15 - 51.81)*	5.63 (4.30 - 6.95)*	7.71 (5.69 - 9.78)*	0.06 (0.00 - 0.12)*	0.17 (0.09 - 0.25)*	2.05 (0.99 - 3.10)*	6.75 (5.60 - 7.90)*	3.27 (2.44 - 4.10)*	-0.17 (-2.13 - 1.79)
Cholesterol in small HDL	SHDLC	6.50 (-6.86 - 19.85)	0.60 (-0.63 - 1.82)	-0.60 (-2.41 - 1.24	-0.02 (-0.08 - 0.03	-0.09 (-0.17 - -0.0	0.47 (-0.66 - 1.59)	1.89 (1.10 - 2.69)*	3.08 (2.19 - 3.97)*	0.64 (-1.31 - 2.59)
Cholesteryl esters in small HDL	SHDLCE	2.64 (-10.71 - 15.99)	-0.09 (-1.32 - 1.14	-1.61 (-3.43 - 0.25	-0.03 (-0.09 - 0.02	-0.12 (-0.20 - -0.0	0.30 (-0.84 - 1.43)	1.13 (0.41 - 1.85)*	2.88 (2.02 - 3.74)*	0.98 (-0.99 - 2.94)
Free cholesterol in small HDL	SHDLFC	25.49 (12.38 - 38.59)*	4.18 (2.86 - 5.49)*	5.34 (3.39 - 7.34)*	0.05 (-0.01 - 0.11)	0.13 (0.05 - 0.20)*	1.23 (0.16 - 2.31)*	5.46 (4.34 - 6.58)*	3.15 (2.32 - 3.99)*	-1.44 (-3.38 - 0.50)
Triglycerides in small HDL	SHDLTG	31.18 (18.41 - 43.96)*	3.69 (2.51 - 4.87)*	4.48 (2.65 - 6.34)*	-0.34 (-0.39 - -0.2	-0.28 (-0.36 - -0.2	2.43 (1.18 - 3.69)*	8.15 (7.32 - 8.98)*	3.27 (2.57 - 3.97)*	6.96 (4.74 - 9.18)*

In the adjusted model for age and sex, differences in the total population between HDL composition and levels of coagulation parameters were observed. The effect size and 95% confidence intervals are presented. After multiple testing correction, significant associations are highlighted with an asterisk (*).

Table S7. Associations between HDL composition and levels of coagulation parameters including ETP, peak, velocity, lag time and time to peak, FVIII, FIX, FXI, and fibrinogen in the total population (model 3).

HDL	Abbreviation	ETP	Peak height	Velocity	Lag time	Time to peak	FVIII	FIX	FXI	Fibrinogen
**Lipoprotein particle sizes**										
Average diameter for HDL particles	HDLD	-14.37 (-28.62 - -0.11)	-1.42 (-2.83 - -0.02	-1.64 (-3.83 - 0.60)	0.30 (0.23 - 0.37)*	0.27 (0.18 - 0.37)*	-0.42 (-1.77 - 0.93)	-4.63 (-5.32 - -3.95	-1.99 (-2.84 - -1.15	-5.28 (-7.27 - -3.29)*
**Lipoprotein particle concentrations**										
Total cholesterol in HDL	HDLC	-1.09 (-15.30 - 13.13)	0.42 (-0.97 - 1.80)	-0.08 (-2.28 - 2.18)	0.24 (0.17 - 0.30)*	0.20 (0.11 - 0.30)*	0.37 (-0.95 - 1.70)	-0.86 (-1.57 - -0.15	1.10 (0.33 - 1.87)*	-4.13 (-6.22 - -2.05)*
Total cholesterol in HDL2	HDL2C	-0.20 (-14.42 - 14.02)	0.51 (-0.88 - 1.89)	0.18 (-2.03 - 2.44)	0.25 (0.18 - 0.32)*	0.23 (0.13 - 0.32)*	0.36 (-0.97 - 1.69)	-1.06 (-1.77 - -0.34	0.88 (0.11 - 1.66)*	-4.28 (-6.36 - -2.19)*
Total cholesterol in HDL3	HDL3C	-8.87 (-22.65 - 4.90)	-0.49 (-1.83 - 0.84)	-2.34 (-4.38 - -0.26	0.05 (-0.01 - 0.12)	-0.03 (-0.12 - 0.06)	0.39 (-0.86 - 1.65)	1.07 (0.42 - 1.73)*	2.82 (2.05 - 3.60)*	-2.05 (-4.00 - -0.10)
Total triglycerides in HDL	HDLTG	-2.88 (-14.81 - 9.06)	0.74 (-0.38 - 1.87)	0.84 (-0.89 - 2.61)	-0.16 (-0.21 - -0.10	-0.12 (-0.19 - -0.05	1.51 (0.24 - 2.79)*	4.04 (3.39 - 4.69)*	1.36 (0.67 - 2.06)*	-3.95 (-5.67 - -2.23)*
**Apolipoproteins**										
Apolipoprotein A1	ApoA1	-6.30 (-20.40 - 7.80)	-0.24 (-1.64 - 1.16)	-1.56 (-3.71 - 0.63)	0.09 (0.02 - 0.16)*	0.02 (-0.07 - 0.11)	1.13 (-0.17 - 2.44)	1.68 (0.99 - 2.38)*	2.99 (2.22 - 3.75)*	-4.16 (-6.20 - -2.11)*
**Very large HDL (average diameter 14.3 nm)**										
Concentration of very large HDL particles	XLHDLP	-21.20 (-35.00 - -7.41)*	-2.16 (-3.62 - -0.71	-2.94 (-5.08 - -0.76	0.18 (0.12 - 0.25)*	0.13 (0.04 - 0.22)*	-0.03 (-1.34 - 1.28)	-3.51 (-4.19 - -2.82	-1.22 (-2.04 - -0.39	-4.19 (-6.02 - -2.35)*
Total lipids in very large HDL	XLHDLL	-21.74 (-35.51 - -7.96)*	-2.21 (-3.66 - -0.76	-3.01 (-5.14 - -0.83	0.18 (0.11 - 0.24)*	0.13 (0.04 - 0.22)*	-0.05 (-1.36 - 1.26)	-3.50 (-4.18 - -2.81	-1.19 (-2.02 - -0.37	-4.17 (-6.01 - -2.34)*
Phospholipids in very large HDL	XLHDLPL	-15.22 (-29.20 - -1.23)*	-1.47 (-2.94 - -0.00	-1.83 (-4.03 - 0.41)	0.23 (0.17 - 0.30)*	0.20 (0.11 - 0.30)*	-0.03 (-1.35 - 1.30)	-3.74 (-4.45 - -3.04	-1.43 (-2.26 - -0.60	-4.28 (-6.16 - -2.41)*
Cholesterol in very large HDL	XLHDLC	-28.06 (-41.52 - -14.61)	-2.95 (-4.36 - -1.54	-4.16 (-6.20 - -2.07	0.12 (0.06 - 0.18)*	0.06 (-0.03 - 0.14)	-0.15 (-1.43 - 1.14)	-3.24 (-3.90 - -2.58	-0.90 (-1.72 - -0.08	-3.79 (-5.59 - -1.99)*
Cholesteryl esters in very large HDL	XLHDLCE	-28.67 (-42.07 - -15.27)	-3.13 (-4.54 - -1.72	-4.48 (-6.50 - -2.41	0.10 (0.04 - 0.16)*	0.03 (-0.06 - 0.12)	-0.14 (-1.42 - 1.14)	-3.26 (-3.92 - -2.61	-0.87 (-1.69 - -0.06	-3.48 (-5.28 - -1.68)*
Free cholesterol in very large HDL	XLHDLFC	-26.07 (-39.66 - -12.48)	-2.46 (-3.87 - -1.04	-3.31 (-5.41 - -1.16	0.16 (0.09 - 0.22)*	0.12 (0.03 - 0.21)*	-0.17 (-1.47 - 1.12)	-3.13 (-3.80 - -2.45	-0.96 (-1.78 - -0.14	-4.46 (-6.26 - -2.65)*
Triglycerides in very large HDL	XLHDLTG	-22.11 (-34.78 - -9.45)*	-2.28 (-3.53 - -1.03	-3.70 (-5.53 - -1.84	-0.08 (-0.13 - -0.02	-0.13 (-0.21 - -0.05	0.83 (-0.35 - 2.01)	0.17 (-0.52 - 0.85)	-0.18 (-0.92 - 0.57)	-3.50 (-5.32 - -1.67)*
**Large HDL (average diameter 12.1 nm)**										
Concentration of large HDL particles	LHDLP	-1.98 (-16.14 - 12.18)	0.23 (-1.12 - 1.58)	0.26 (-1.90 - 2.47)	0.31 (0.25 - 0.38)*	0.30 (0.21 - 0.39)*	0.21 (-1.13 - 1.54)	-2.58 (-3.28 - -1.89	-0.98 (-1.78 - -0.18	-5.44 (-7.37 - -3.51)*
Total lipids in large HDL	LHDLL	-2.23 (-16.38 - 11.92)	0.21 (-1.14 - 1.56)	0.24 (-1.92 - 2.46)	0.31 (0.25 - 0.38)*	0.30 (0.21 - 0.39)*	0.18 (-1.16 - 1.52)	-2.64 (-3.33 - -1.94	-1.01 (-1.80 - -0.21	-5.44 (-7.36 - -3.51)*
Phospholipids in large HDL	LHDLPL	-0.10 (-14.31 - 14.10)	0.49 (-0.85 - 1.84)	0.48 (-1.68 - 2.68)	0.31 (0.24 - 0.38)*	0.30 (0.21 - 0.39)*	0.34 (-1.00 - 1.68)	-2.04 (-2.74 - -1.34	-0.52 (-1.31 - 0.28)	-5.24 (-7.19 - -3.29)*
Cholesterol in large HDL	LHDLC	-4.08 (-18.12 - 9.95)	-0.03 (-1.38 - 1.32)	0.07 (-2.09 - 2.28)	0.31 (0.25 - 0.38)*	0.30 (0.22 - 0.39)*	0.00 (-1.33 - 1.33)	-3.10 (-3.79 - -2.40	-1.35 (-2.14 - -0.55	-5.47 (-7.37 - -3.56)*
Cholesteryl esters in large HDL	LHDLCE	-3.97 (-18.01 - 10.07)	-0.03 (-1.38 - 1.32)	0.08 (-2.08 - 2.29)	0.31 (0.25 - 0.38)*	0.31 (0.22 - 0.40)*	0.00 (-1.33 - 1.33)	-3.10 (-3.80 - -2.41	-1.38 (-2.17 - -0.58	-5.51 (-7.42 - -3.60)*
Free cholesterol in large HDL	LHDLFC	-4.42 (-18.44 - 9.60)	-0.04 (-1.39 - 1.30)	0.04 (-2.12 - 2.25)	0.30 (0.24 - 0.37)*	0.30 (0.21 - 0.39)*	0.00 (-1.33 - 1.33)	-3.07 (-3.77 - -2.38	-1.25 (-2.05 - -0.45	-5.32 (-7.23 - -3.41)*
Triglycerides in large HDL	LHDLTG	-0.72 (-14.55 - 13.11)	0.01 (-1.34 - 1.36)	-0.23 (-2.31 - 1.90)	0.22 (0.16 - 0.28)*	0.20 (0.12 - 0.28)*	0.70 (-0.54 - 1.95)	-2.28 (-2.96 - -1.60	-1.74 (-2.52 - -0.96	-5.32 (-7.15 - -3.48)*
**Medium HDL (average diameter 10.9 nm)**										
Concentration of medium HDL particles	MHDLP	16.91 (3.98 - 29.85)*	3.17 (1.92 - 4.41)*	3.86 (1.88 - 5.87)*	0.15 (0.09 - 0.21)*	0.19 (0.11 - 0.28)*	1.39 (0.21 - 2.57)*	3.91 (3.23 - 4.59)*	3.00 (2.20 - 3.79)*	-5.24 (-7.15 - -3.34)*
Total lipids in medium HDL	MHDLL	16.75 (3.78 - 29.73)*	3.10 (1.86 - 4.35)*	3.76 (1.78 - 5.78)*	0.16 (0.09 - 0.22)*	0.20 (0.11 - 0.28)*	1.32 (0.14 - 2.51)*	3.72 (3.04 - 4.40)*	2.93 (2.14 - 3.73)*	-5.16 (-7.07 - -3.25)*
Phospholipids in medium HDL	MHDLPL	16.51 (3.41 - 29.62)*	3.25 (1.99 - 4.52)*	3.97 (1.96 - 6.02)*	0.15 (0.09 - 0.21)*	0.20 (0.12 - 0.28)*	1.36 (0.16 - 2.55)*	3.81 (3.13 - 4.48)*	2.84 (2.05 - 3.63)*	-5.42 (-7.31 - -3.53)*
Cholesterol in medium HDL	MHDLC	15.83 (2.87 - 28.79)*	2.68 (1.46 - 3.91)*	3.18 (1.23 - 5.18)*	0.18 (0.12 - 0.25)*	0.21 (0.12 - 0.29)*	1.00 (-0.19 - 2.18)	2.81 (2.13 - 3.49)*	2.63 (1.83 - 3.44)*	-4.58 (-6.53 - -2.62)*
Cholesteryl esters in medium HDL	MHDLCE	15.82 (2.93 - 28.70)*	2.63 (1.41 - 3.85)*	3.15 (1.21 - 5.13)*	0.19 (0.12 - 0.25)*	0.21 (0.12 - 0.29)*	0.96 (-0.21 - 2.14)	2.74 (2.06 - 3.41)*	2.59 (1.79 - 3.39)*	-4.52 (-6.48 - -2.57)*
Free cholesterol in medium HDL	MHDLFC	15.61 (2.37 - 28.84)*	2.84 (1.57 - 4.10)*	3.25 (1.24 - 5.30)*	0.17 (0.11 - 0.24)*	0.20 (0.12 - 0.29)*	1.11 (-0.10 - 2.32)	3.02 (2.34 - 3.70)*	2.75 (1.96 - 3.54)*	-4.70 (-6.64 - -2.76)*
Triglycerides in medium HDL	MHDLTG	7.31 (-4.69 - 19.31)	2.04 (0.86 - 3.23)*	2.72 (0.90 - 4.57)*	-0.18 (-0.23 - -0.12	-0.11 (-0.18 - -0.03	2.06 (0.82 - 3.31)*	5.87 (5.18 - 6.55)*	2.57 (1.87 - 3.27)*	-2.50 (-4.19 - -0.81)*
**Small HDL (average diameter 8.7 nm)**										
Concentration of small HDL particles	SHDLP	21.48 (9.49 - 33.47)*	3.25 (2.07 - 4.43)*	3.54 (1.79 - 5.31)*	0.00 (-0.05 - 0.06)	0.03 (-0.05 - 0.10)	1.27 (0.18 - 2.36)*	4.81 (3.80 - 5.83)*	3.57 (2.67 - 4.48)*	-0.62 (-2.35 - 1.11)
Total lipids in small HDL	SHDLL	21.02 (9.00 - 33.03)*	3.14 (1.96 - 4.31)*	3.35 (1.62 - 5.12)*	0.01 (-0.05 - 0.07)	0.03 (-0.04 - 0.11)	1.21 (0.12 - 2.30)*	4.59 (3.61 - 5.58)*	3.52 (2.62 - 4.42)*	-0.51 (-2.25 - 1.22)
Phospholipids in small HDL	SHDLPL	26.62 (14.49 - 38.75)*	4.56 (3.31 - 5.81)*	6.26 (4.34 - 8.21)*	0.09 (0.04 - 0.15)*	0.19 (0.12 - 0.27)*	1.30 (0.17 - 2.42)*	5.47 (4.45 - 6.49)*	2.85 (2.02 - 3.69)*	-3.56 (-5.48 - -1.64)*
Cholesterol in small HDL	SHDLC	12.57 (0.19 - 24.95)	1.26 (0.11 - 2.40)*	0.29 (-1.45 - 2.05)	-0.03 (-0.08 - 0.03)	-0.08 (-0.15 - -0.00	0.80 (-0.33 - 1.93)	2.36 (1.66 - 3.05)*	3.16 (2.28 - 4.04)*	2.12 (0.41 - 3.83)*
Cholesteryl esters in small HDL	SHDLCE	10.67 (-1.81 - 23.15)	0.77 (-0.38 - 1.93)	-0.47 (-2.23 - 1.32)	-0.04 (-0.10 - 0.01)	-0.11 (-0.19 - -0.04	0.76 (-0.39 - 1.90)	1.82 (1.17 - 2.47)*	3.05 (2.19 - 3.91)*	2.95 (1.23 - 4.68)*
Free cholesterol in small HDL	SHDLFC	19.66 (7.43 - 31.89)*	3.62 (2.41 - 4.83)*	4.56 (2.73 - 6.42)*	0.07 (0.01 - 0.12)*	0.13 (0.05 - 0.21)*	0.80 (-0.34 - 1.94)	4.70 (3.74 - 5.65)*	2.91 (2.08 - 3.74)*	-3.10 (-5.00 - -1.20)*
Triglycerides in small HDL	SHDLTG	2.55 (-9.96 - 15.06)	1.47 (0.27 - 2.68)*	1.69 (-0.19 - 3.61)	-0.26 (-0.32 - -0.20	-0.20 (-0.29 - -0.12	1.05 (-0.25 - 2.36)	5.51 (4.74 - 6.27)*	2.17 (1.45 - 2.88)*	-0.76 (-2.69 - 1.18)

In the adjusted model for age, sex, race, menopausal status, lipid-lowering drugs, C-reactive protein, total body fat, and body mass index, differences in the total population between HDL composition and levels of coagulation parameters were observed. The effect size and 95% confidence intervals are presented. After multiple testing correction, significant associations are highlighted with an asterisk (*).

Table S8. Associations between HDL composition and platelet activation parameters including platelet count, mean platelet volume, and platelet distribution width in the total population (model 1).

HDL	Abbreviation	Platelet count	Mean platelet volume	Platelet distribution width
**Lipoprotein particle sizes**				
Average diameter for HDL particles	HDLD	6.41 (3.11 - 9.71)*	-0.01 (-0.06 - 0.04)	-0.09 (-0.20 - 0.01)
**Lipoprotein particle concentrations**				
Total cholesterol in HDL	HDLC	8.38 (5.08 - 11.69)*	-0.03 (-0.08 - 0.02)	-0.13 (-0.24 - -0.03)*
Total cholesterol in HDL2	HDL2C	8.27 (4.96 - 11.57)*	-0.03 (-0.08 - 0.02)	-0.13 (-0.24 - -0.03)*
Total cholesterol in HDL3	HDL3C	8.44 (5.07 - 11.81)*	-0.04 (-0.09 - 0.02)	-0.13 (-0.24 - -0.02)
Total triglycerides in HDL	HDLTG	3.30 (-0.54 - 7.15)	0.04 (-0.01 - 0.10)	0.09 (-0.01 - 0.19)
**Apolipoproteins**				
Apolipoprotein A1	ApoA1	9.66 (6.45 - 12.88)*	-0.04 (-0.09 - 0.01)	-0.14 (-0.25 - -0.03)*
**Very large HDL (average diameter 14.3 nm)**				
Concentration of very large HDL particles	XLHDLP	6.31 (3.14 - 9.48)*	-0.01 (-0.05 - 0.04)	-0.08 (-0.18 - 0.02)
Total lipids in very large HDL	XLHDLL	6.30 (3.13 - 9.46)*	-0.01 (-0.06 - 0.04)	-0.08 (-0.18 - 0.01)
Phospholipids in very large HDL	XLHDLPL	6.32 (3.14 - 9.50)*	-0.00 (-0.05 - 0.04)	-0.07 (-0.17 - 0.03)
Cholesterol in very large HDL	XLHDLC	6.09 (2.92 - 9.25)*	-0.02 (-0.06 - 0.03)	-0.10 (-0.19 - -0.00)
Cholesteryl esters in very large HDL	XLHDLCE	5.97 (2.78 - 9.17)*	-0.02 (-0.07 - 0.02)	-0.10 (-0.20 - -0.00)
Free cholesterol in very large HDL	XLHDLFC	6.29 (3.19 - 9.40)*	-0.01 (-0.06 - 0.03)	-0.09 (-0.19 - 0.00)
Triglycerides in very large HDL	XLHDLTG	5.55 (1.86 - 9.25)*	-0.00 (-0.05 - 0.05)	-0.03 (-0.13 - 0.08)
**Large HDL (average diameter 12.1 nm)**				
Concentration of large HDL particles	LHDLP	7.00 (3.60 - 10.40)*	-0.01 (-0.06 - 0.03)	-0.09 (-0.20 - 0.01)
Total lipids in large HDL	LHDLL	6.95 (3.57 - 10.34)*	-0.01 (-0.06 - 0.03)	-0.09 (-0.20 - 0.01)
Phospholipids in large HDL	LHDLPL	7.27 (3.75 - 10.80)*	-0.02 (-0.07 - 0.03)	-0.11 (-0.21 - 0.00)
Cholesterol in large HDL	LHDLC	6.61 (3.34 - 9.87)*	-0.01 (-0.06 - 0.04)	-0.09 (-0.19 - 0.02)
Cholesteryl esters in large HDL	LHDLCE	6.62 (3.34 - 9.90)*	-0.01 (-0.06 - 0.04)	-0.09 (-0.19 - 0.02)
Free cholesterol in large HDL	LHDLFC	6.56 (3.33 - 9.78)*	-0.01 (-0.06 - 0.04)	-0.08 (-0.19 - 0.02)
Triglycerides in large HDL	LHDLTG	6.84 (3.43 - 10.25)*	0.00 (-0.05 - 0.05)	-0.05 (-0.16 - 0.05)
**Medium HDL (average diameter 10.9 nm)**				
Concentration of medium HDL particles	MHDLP	8.41 (4.84 - 11.99)*	-0.03 (-0.09 - 0.02)	-0.11 (-0.22 - 0.01)
Total lipids in medium HDL	MHDLL	8.37 (4.78 - 11.95)*	-0.03 (-0.09 - 0.02)	-0.11 (-0.22 - 0.01)
Phospholipids in medium HDL	MHDLPL	8.50 (4.95 - 12.05)*	-0.03 (-0.08 - 0.03)	-0.10 (-0.22 - 0.01)
Cholesterol in medium HDL	MHDLC	7.86 (4.24 - 11.48)*	-0.04 (-0.09 - 0.02)	-0.12 (-0.23 - 0.00)
Cholesteryl esters in medium HDL	MHDLCE	7.68 (4.07 - 11.28)*	-0.04 (-0.09 - 0.02)	-0.11 (-0.23 - 0.00)
Free cholesterol in medium HDL	MHDLFC	8.42 (4.77 - 12.06)*	-0.04 (-0.09 - 0.02)	-0.12 (-0.24 - -0.01)
Triglycerides in medium HDL	MHDLTG	1.95 (-1.68 - 5.57)	0.02 (-0.03 - 0.07)	0.06 (-0.04 - 0.17)
**Small HDL (average diameter 8.7 nm)**				
Concentration of small HDL particles	SHDLP	4.14 (0.38 - 7.90)	-0.03 (-0.08 - 0.03)	-0.07 (-0.18 - 0.05)
Total lipids in small HDL	SHDLL	4.24 (0.47 - 8.01)	-0.03 (-0.09 - 0.03)	-0.07 (-0.19 - 0.04)
Phospholipids in small HDL	SHDLPL	4.05 (0.33 - 7.77)	-0.01 (-0.06 - 0.05)	-0.03 (-0.14 - 0.09)
Cholesterol in small HDL	SHDLC	4.05 (0.36 - 7.74)	-0.05 (-0.11 - 0.01)	-0.12 (-0.24 - 0.00)
Cholesteryl esters in small HDL	SHDLCE	3.88 (0.24 - 7.53)	-0.05 (-0.11 - 0.00)	-0.12 (-0.24 - -0.01)
Free cholesterol in small HDL	SHDLFC	3.82 (0.12 - 7.53)	-0.01 (-0.07 - 0.04)	-0.04 (-0.16 - 0.07)
Triglycerides in small HDL	SHDLTG	-3.00 (-6.36 - 0.35)	0.05 (0.00 - 0.11)	0.15 (0.05 - 0.26)*

In the crude model, differences in the total population between HDL composition and platelet activation parameters were observed. The effect size and 95% confidence intervals are presented. After multiple testing correction, significant associations are highlighted with an asterisk (*).

Table S9. Associations between HDL composition and platelet activation parameters including platelet count, mean platelet volume, and platelet distribution width in the total population (model 2).

HDL	Abbreviation	Platelet count	Mean platelet volume	Platelet distribution width
**Lipoprotein particle sizes**				
Average diameter for HDL particles	HDLD	-1.45 (-5.26 - 2.36)	-0.01 (-0.06 - 0.05)	-0.09 (-0.20 - 0.03)
**Lipoprotein particle concentrations**				
Total cholesterol in HDL	HDLC	1.69 (-1.93 - 5.31)	-0.04 (-0.09 - 0.02)	-0.14 (-0.26 - -0.02)
Total cholesterol in HDL2	HDL2C	1.46 (-2.19 - 5.10)	-0.04 (-0.09 - 0.02)	-0.14 (-0.26 - -0.02)
Total cholesterol in HDL3	HDL3C	3.58 (0.23 - 6.92)	-0.04 (-0.10 - 0.02)	-0.13 (-0.25 - -0.00)
Total triglycerides in HDL	HDLTG	2.15 (-1.34 - 5.65)	0.05 (-0.01 - 0.10)	0.10 (-0.01 - 0.20)
**Apolipoproteins**				
Apolipoprotein A1	ApoA1	3.97 (0.52 - 7.42)	-0.04 (-0.10 - 0.02)	-0.14 (-0.27 - -0.02)
**Very large HDL (average diameter 14.3 nm)**				
Concentration of very large HDL particles	XLHDLP	-0.51 (-4.20 - 3.17)	-0.01 (-0.06 - 0.04)	-0.07 (-0.19 - 0.04)
Total lipids in very large HDL	XLHDLL	-0.52 (-4.20 - 3.17)	-0.01 (-0.06 - 0.04)	-0.08 (-0.19 - 0.03)
Phospholipids in very large HDL	XLHDLPL	-0.82 (-4.52 - 2.89)	-0.00 (-0.05 - 0.05)	-0.06 (-0.18 - 0.05)
Cholesterol in very large HDL	XLHDLC	-0.31 (-3.94 - 3.33)	-0.02 (-0.07 - 0.03)	-0.09 (-0.20 - 0.01)
Cholesteryl esters in very large HDL	XLHDLCE	-0.23 (-3.87 - 3.40)	-0.02 (-0.07 - 0.03)	-0.09 (-0.20 - 0.01)
Free cholesterol in very large HDL	XLHDLFC	-0.47 (-4.11 - 3.16)	-0.02 (-0.07 - 0.03)	-0.09 (-0.20 - 0.02)
Triglycerides in very large HDL	XLHDLTG	1.86 (-1.77 - 5.50)	-0.00 (-0.05 - 0.05)	-0.01 (-0.12 - 0.10)
**Large HDL (average diameter 12.1 nm)**				
Concentration of large HDL particles	LHDLP	-0.22 (-4.15 - 3.70)	-0.02 (-0.07 - 0.04)	-0.09 (-0.21 - 0.03)
Total lipids in large HDL	LHDLL	-0.27 (-4.17 - 3.63)	-0.01 (-0.07 - 0.04)	-0.09 (-0.21 - 0.03)
Phospholipids in large HDL	LHDLPL	0.08 (-3.94 - 4.10)	-0.02 (-0.08 - 0.03)	-0.10 (-0.23 - 0.02)
Cholesterol in large HDL	LHDLC	-0.60 (-4.38 - 3.19)	-0.01 (-0.06 - 0.04)	-0.08 (-0.19 - 0.04)
Cholesteryl esters in large HDL	LHDLCE	-0.58 (-4.38 - 3.21)	-0.01 (-0.06 - 0.04)	-0.08 (-0.19 - 0.04)
Free cholesterol in large HDL	LHDLFC	-0.63 (-4.36 - 3.10)	-0.01 (-0.06 - 0.04)	-0.08 (-0.19 - 0.04)
Triglycerides in large HDL	LHDLTG	0.36 (-3.48 - 4.21)	0.01 (-0.05 - 0.06)	-0.03 (-0.15 - 0.09)
**Medium HDL (average diameter 10.9 nm)**				
Concentration of medium HDL particles	MHDLP	3.52 (0.01 - 7.04)	-0.03 (-0.09 - 0.03)	-0.10 (-0.22 - 0.03)
Total lipids in medium HDL	MHDLL	3.44 (-0.08 - 6.96)	-0.03 (-0.09 - 0.03)	-0.10 (-0.23 - 0.03)
Phospholipids in medium HDL	MHDLPL	3.42 (-0.11 - 6.95)	-0.03 (-0.09 - 0.03)	-0.09 (-0.22 - 0.03)
Cholesterol in medium HDL	MHDLC	2.97 (-0.52 - 6.47)	-0.04 (-0.10 - 0.02)	-0.11 (-0.24 - 0.02)
Cholesteryl esters in medium HDL	MHDLCE	2.87 (-0.60 - 6.34)	-0.04 (-0.10 - 0.02)	-0.11 (-0.23 - 0.02)
Free cholesterol in medium HDL	MHDLFC	3.31 (-0.27 - 6.89)	-0.04 (-0.10 - 0.02)	-0.12 (-0.25 - 0.01)
Triglycerides in medium HDL	MHDLTG	3.06 (-0.39 - 6.50)	0.02 (-0.03 - 0.07)	0.06 (-0.04 - 0.17)
**Small HDL (average diameter 8.7 nm)**				
Concentration of small HDL particles	SHDLP	4.03 (0.36 - 7.69)	-0.03 (-0.08 - 0.03)	-0.06 (-0.18 - 0.05)
Total lipids in small HDL	SHDLL	3.99 (0.33 - 7.65)	-0.03 (-0.08 - 0.03)	-0.07 (-0.19 - 0.05)
Phospholipids in small HDL	SHDLPL	3.18 (-0.37 - 6.73)	-0.01 (-0.06 - 0.05)	-0.02 (-0.14 - 0.09)
Cholesterol in small HDL	SHDLC	3.73 (0.20 - 7.26)	-0.05 (-0.11 - 0.01)	-0.11 (-0.23 - 0.01)
Cholesteryl esters in small HDL	SHDLCE	3.72 (0.25 - 7.20)	-0.05 (-0.11 - 0.01)	-0.12 (-0.24 - -0.00)
Free cholesterol in small HDL	SHDLFC	2.66 (-0.87 - 6.18)	-0.01 (-0.07 - 0.05)	-0.03 (-0.15 - 0.08)
Triglycerides in small HDL	SHDLTG	1.01 (-2.46 - 4.48)	0.06 (0.01 - 0.11)	0.15 (0.05 - 0.26)

In the adjusted model for age and sex, differences in the total population between HDL composition and platelet activation parameters were observed. The effect size and 95% confidence intervals are presented. After multiple testing correction, significant associations are highlighted with an asterisk (*).

Table S10. Associations between HDL composition and platelet activation parameters including platelet count, mean platelet volume, and platelet distribution width in the total population (model 3).

HDL	Abbreviation	Platelet count	Mean platelet volume	Platelet distribution width
**Lipoprotein particle sizes**				
Average diameter for HDL particles	HDLD	-0.76 (-5.02 - 3.50)	0.01 (-0.05 - 0.07)	-0.05 (-0.18 - 0.08)
**Lipoprotein particle concentrations**				
Total cholesterol in HDL	HDLC	2.74 (-1.15 - 6.62)	-0.03 (-0.09 - 0.04)	-0.11 (-0.25 - 0.02)
Total cholesterol in HDL2	HDL2C	2.49 (-1.44 - 6.42)	-0.03 (-0.09 - 0.04)	-0.11 (-0.24 - 0.02)
Total cholesterol in HDL3	HDL3C	4.38 (0.99 - 7.76)	-0.03 (-0.09 - 0.03)	-0.10 (-0.23 - 0.03)
Total triglycerides in HDL	HDLTG	1.38 (-2.21 - 4.96)	0.05 (-0.01 - 0.10)	0.09 (-0.02 - 0.20)
**Apolipoproteins**				
Apolipoprotein A1	ApoA1	4.86 (1.29 - 8.43)	-0.03 (-0.10 - 0.03)	-0.11 (-0.24 - 0.02)
**Very large HDL (average diameter 14.3 nm)**				
Concentration of very large HDL particles	XLHDLP	0.16 (-3.86 - 4.18)	0.01 (-0.05 - 0.06)	-0.04 (-0.16 - 0.08)
Total lipids in very large HDL	XLHDLL	0.16 (-3.85 - 4.18)	0.00 (-0.05 - 0.06)	-0.04 (-0.16 - 0.08)
Phospholipids in very large HDL	XLHDLPL	-0.16 (-4.21 - 3.89)	0.01 (-0.04 - 0.07)	-0.03 (-0.15 - 0.09)
Cholesterol in very large HDL	XLHDLC	0.40 (-3.55 - 4.35)	-0.01 (-0.06 - 0.05)	-0.06 (-0.17 - 0.06)
Cholesteryl esters in very large HDL	XLHDLCE	0.48 (-3.46 - 4.42)	-0.01 (-0.06 - 0.05)	-0.06 (-0.17 - 0.06)
Free cholesterol in very large HDL	XLHDLFC	0.21 (-3.75 - 4.16)	-0.00 (-0.06 - 0.05)	-0.05 (-0.17 - 0.06)
Triglycerides in very large HDL	XLHDLTG	1.85 (-1.80 - 5.51)	0.01 (-0.04 - 0.06)	0.01 (-0.10 - 0.13)
**Large HDL (average diameter 12.1 nm)**				
Concentration of large HDL particles	LHDLP	0.46 (-3.74 - 4.66)	-0.01 (-0.06 - 0.05)	-0.06 (-0.19 - 0.06)
Total lipids in large HDL	LHDLL	0.42 (-3.77 - 4.60)	-0.01 (-0.06 - 0.05)	-0.06 (-0.19 - 0.06)
Phospholipids in large HDL	LHDLPL	0.78 (-3.49 - 5.05)	-0.01 (-0.07 - 0.04)	-0.08 (-0.21 - 0.05)
Cholesterol in large HDL	LHDLC	0.08 (-4.01 - 4.17)	0.00 (-0.06 - 0.06)	-0.05 (-0.17 - 0.07)
Cholesteryl esters in large HDL	LHDLCE	0.09 (-4.02 - 4.19)	0.00 (-0.06 - 0.06)	-0.05 (-0.17 - 0.07)
Free cholesterol in large HDL	LHDLFC	0.05 (-3.99 - 4.10)	0.00 (-0.06 - 0.06)	-0.05 (-0.17 - 0.07)
Triglycerides in large HDL	LHDLTG	0.71 (-3.34 - 4.76)	0.02 (-0.04 - 0.08)	-0.00 (-0.13 - 0.12)
**Medium HDL (average diameter 10.9 nm)**				
Concentration of medium HDL particles	MHDLP	3.78 (0.26 - 7.29)	-0.03 (-0.09 - 0.03)	-0.09 (-0.22 - 0.04)
Total lipids in medium HDL	MHDLL	3.73 (0.21 - 7.26)	-0.03 (-0.10 - 0.03)	-0.09 (-0.23 - 0.04)
Phospholipids in medium HDL	MHDLPL	3.64 (0.10 - 7.18)	-0.03 (-0.09 - 0.03)	-0.09 (-0.22 - 0.04)
Cholesterol in medium HDL	MHDLC	3.48 (-0.04 - 7.00)	-0.04 (-0.10 - 0.03)	-0.10 (-0.23 - 0.03)
Cholesteryl esters in medium HDL	MHDLCE	3.38 (-0.11 - 6.88)	-0.04 (-0.10 - 0.03)	-0.10 (-0.23 - 0.03)
Free cholesterol in medium HDL	MHDLFC	3.77 (0.17 - 7.38)	-0.04 (-0.10 - 0.03)	-0.11 (-0.24 - 0.02)
Triglycerides in medium HDL	MHDLTG	2.27 (-1.39 - 5.92)	0.02 (-0.04 - 0.07)	0.04 (-0.07 - 0.15)
**Small HDL (average diameter 8.7 nm)**				
Concentration of small HDL particles	SHDLP	3.97 (0.39 - 7.54)	-0.03 (-0.09 - 0.03)	-0.08 (-0.20 - 0.04)
Total lipids in small HDL	SHDLL	3.97 (0.41 - 7.53)	-0.03 (-0.09 - 0.02)	-0.08 (-0.21 - 0.04)
Phospholipids in small HDL	SHDLPL	2.86 (-0.71 - 6.44)	-0.02 (-0.08 - 0.04)	-0.05 (-0.17 - 0.07)
Cholesterol in small HDL	SHDLC	4.13 (0.70 - 7.56)	-0.05 (-0.10 - 0.01)	-0.11 (-0.23 - 0.01)
Cholesteryl esters in small HDL	SHDLCE	4.22 (0.83 - 7.61)	-0.05 (-0.11 - 0.01)	-0.11 (-0.23 - 0.00)
Free cholesterol in small HDL	SHDLFC	2.54 (-0.93 - 6.01)	-0.02 (-0.08 - 0.04)	-0.05 (-0.18 - 0.07)
Triglycerides in small HDL	SHDLTG	-0.11 (-3.85 - 3.63)	0.05 (-0.00 - 0.11)	0.13 (0.02 - 0.24)

In the adjusted model for age, sex, race, menopausal status, lipid-lowering drugs, C-reactive protein, total body fat, and body mass index, differences in the total population between HDL composition and platelet activation parameters were observed. The effect size and 95% confidence intervals are presented. After multiple testing correction, significant associations are highlighted with an asterisk (*).

Table S11. Associations between HDL composition and levels of glycocalyx-related parameters including PBRTotal, PBRfeed vessel, and PBRcapillary in the total population (model 1)

HDL	Abbreviation	PBR total	PBR feed vessel	PBR capillary
**Lipoprotein particle sizes**				
Average diameter for HDL particles	HDLD	0.02 (0.00 - 0.04)	0.03 (0.00 - 0.05)	0.00 (-0.00 - 0.01)
**Lipoprotein particle concentrations**				
Total cholesterol in HDL	HDLC	0.03 (0.00 - 0.05)	0.03 (0.01 - 0.06)	0.01 (-0.00 - 0.01)
Total cholesterol in HDL2	HDL2C	0.03 (0.00 - 0.05)	0.03 (0.01 - 0.06)	0.01 (-0.00 - 0.01)
Total cholesterol in HDL3	HDL3C	0.02 (-0.01 - 0.04)	0.03 (0.00 - 0.05)	0.00 (-0.01 - 0.01)
Total triglycerides in HDL	HDLTG	0.00 (-0.02 - 0.02)	-0.00 (-0.03 - 0.02)	-0.01 (-0.02 - -0.00)
**Apolipoproteins**				
Apolipoprotein A1	ApoA1	0.03 (0.00 - 0.05)	0.03 (0.01 - 0.06)	0.00 (-0.01 - 0.01)
**Very large HDL (average diameter 14.3 nm)**				
Concentration of very large HDL particles	XLHDLP	0.02 (-0.01 - 0.04)	0.02 (-0.01 - 0.04)	0.00 (-0.01 - 0.01)
Total lipids in very large HDL	XLHDLL	0.02 (-0.01 - 0.04)	0.02 (-0.01 - 0.04)	0.00 (-0.01 - 0.01)
Phospholipids in very large HDL	XLHDLPL	0.02 (-0.00 - 0.04)	0.02 (-0.00 - 0.04)	0.00 (-0.00 - 0.01)
Cholesterol in very large HDL	XLHDLC	0.01 (-0.01 - 0.03)	0.01 (-0.01 - 0.04)	0.00 (-0.01 - 0.01)
Cholesteryl esters in very large HDL	XLHDLCE	0.01 (-0.01 - 0.03)	0.01 (-0.01 - 0.03)	0.00 (-0.01 - 0.01)
Free cholesterol in very large HDL	XLHDLFC	0.02 (-0.01 - 0.04)	0.02 (-0.01 - 0.04)	0.00 (-0.01 - 0.01)
Triglycerides in very large HDL	XLHDLTG	-0.00 (-0.02 - 0.02)	-0.01 (-0.03 - 0.02)	-0.01 (-0.01 - 0.00)
**Large HDL (average diameter 12.1 nm)**				
Concentration of large HDL particles	LHDLP	0.02 (0.00 - 0.05)	0.03 (0.00 - 0.05)	0.00 (-0.00 - 0.01)
Total lipids in large HDL	LHDLL	0.02 (0.00 - 0.05)	0.03 (0.00 - 0.05)	0.00 (-0.00 - 0.01)
Phospholipids in large HDL	LHDLPL	0.02 (0.00 - 0.05)	0.03 (0.00 - 0.06)	0.00 (-0.00 - 0.01)
Cholesterol in large HDL	LHDLC	0.02 (0.00 - 0.05)	0.03 (0.00 - 0.05)	0.00 (-0.00 - 0.01)
Cholesteryl esters in large HDL	LHDLCE	0.02 (0.00 - 0.05)	0.03 (0.00 - 0.05)	0.00 (-0.00 - 0.01)
Free cholesterol in large HDL	LHDLFC	0.02 (0.00 - 0.05)	0.03 (0.00 - 0.05)	0.00 (-0.00 - 0.01)
Triglycerides in large HDL	LHDLTG	0.01 (-0.01 - 0.04)	0.01 (-0.01 - 0.04)	0.00 (-0.01 - 0.01)
**Medium HDL (average diameter 10.9 nm)**				
Concentration of medium HDL particles	MHDLP	0.03 (0.00 - 0.05)	0.03 (-0.00 - 0.06)	0.00 (-0.00 - 0.01)
Total lipids in medium HDL	MHDLL	0.03 (0.00 - 0.05)	0.03 (-0.00 - 0.06)	0.01 (-0.00 - 0.01)
Phospholipids in medium HDL	MHDLPL	0.03 (0.00 - 0.05)	0.03 (-0.00 - 0.06)	0.00 (-0.00 - 0.01)
Cholesterol in medium HDL	MHDLC	0.03 (0.00 - 0.05)	0.03 (0.00 - 0.06)	0.01 (-0.00 - 0.02)
Cholesteryl esters in medium HDL	MHDLCE	0.03 (0.00 - 0.05)	0.03 (0.00 - 0.06)	0.01 (-0.00 - 0.02)
Free cholesterol in medium HDL	MHDLFC	0.03 (0.00 - 0.05)	0.03 (0.00 - 0.06)	0.01 (-0.00 - 0.01)
Triglycerides in medium HDL	MHDLTG	0.00 (-0.02 - 0.02)	-0.01 (-0.03 - 0.02)	-0.01 (-0.02 - 0.00)
**Small HDL (average diameter 8.7 nm)**				
Concentration of small HDL particles	SHDLP	0.00 (-0.02 - 0.02)	0.01 (-0.02 - 0.03)	0.00 (-0.00 - 0.01)
Total lipids in small HDL	SHDLL	0.01 (-0.01 - 0.03)	0.01 (-0.02 - 0.04)	0.00 (-0.00 - 0.01)
Phospholipids in small HDL	SHDLPL	0.01 (-0.01 - 0.04)	0.01 (-0.02 - 0.04)	0.01 (-0.00 - 0.01)
Cholesterol in small HDL	SHDLC	0.00 (-0.01 - 0.02)	0.01 (-0.01 - 0.04)	0.00 (-0.00 - 0.01)
Cholesteryl esters in small HDL	SHDLCE	0.00 (-0.02 - 0.02)	0.01 (-0.01 - 0.04)	0.00 (-0.01 - 0.01)
Free cholesterol in small HDL	SHDLFC	0.01 (-0.01 - 0.03)	0.01 (-0.02 - 0.04)	0.01 (-0.00 - 0.01)
Triglycerides in small HDL	SHDLTG	-0.02 (-0.04 - -0.00)	-0.03 (-0.05 - -0.01)	-0.01 (-0.02 - 0.00)

In the crude model, differences in the total population between HDL composition and glycocalyx-related parameters were observed. The effect size and 95% confidence intervals are presented. After multiple testing correction, significant associations are highlighted with an asterisk (*).

Table S12. Associations between HDL composition and levels of glycocalyx-related parameters including PBRTotal, PBRfeed vessel, and PBRcapillary in the total population (model 2)

HDL	Abbreviation	PBR total	PBR feed vessel	PBR capillary
**Lipoprotein particle sizes**				
Average diameter for HDL particles	HDLD	0.00 (-0.02 - 0.03)	0.00 (-0.03 - 0.03)	0.00 (-0.01 - 0.01)
**Lipoprotein particle concentrations**				
Total cholesterol in HDL	HDLC	0.01 (-0.02 - 0.03)	0.01 (-0.02 - 0.04)	0.01 (-0.00 - 0.02)
Total cholesterol in HDL2	HDL2C	0.01 (-0.02 - 0.03)	0.01 (-0.02 - 0.04)	0.01 (-0.00 - 0.02)
Total cholesterol in HDL3	HDL3C	0.00 (-0.02 - 0.02)	0.01 (-0.02 - 0.04)	0.00 (-0.01 - 0.01)
Total triglycerides in HDL	HDLTG	-0.00 (-0.02 - 0.02)	-0.01 (-0.03 - 0.02)	-0.01 (-0.02 - 0.00)
**Apolipoproteins**				
Apolipoprotein A1	ApoA1	0.01 (-0.02 - 0.03)	0.01 (-0.02 - 0.04)	0.00 (-0.01 - 0.01)
**Very large HDL (average diameter 14.3 nm)**				
Concentration of very large HDL particles	XLHDLP	-0.00 (-0.03 - 0.02)	-0.01 (-0.04 - 0.02)	0.00 (-0.01 - 0.01)
Total lipids in very large HDL	XLHDLL	-0.00 (-0.03 - 0.02)	-0.01 (-0.04 - 0.02)	0.00 (-0.01 - 0.01)
Phospholipids in very large HDL	XLHDLPL	-0.00 (-0.03 - 0.02)	-0.01 (-0.03 - 0.02)	0.00 (-0.01 - 0.01)
Cholesterol in very large HDL	XLHDLC	-0.01 (-0.03 - 0.02)	-0.01 (-0.04 - 0.02)	-0.00 (-0.01 - 0.01)
Cholesteryl esters in very large HDL	XLHDLCE	-0.01 (-0.03 - 0.02)	-0.01 (-0.04 - 0.01)	-0.00 (-0.01 - 0.01)
Free cholesterol in very large HDL	XLHDLFC	-0.00 (-0.03 - 0.02)	-0.01 (-0.03 - 0.02)	0.00 (-0.01 - 0.01)
Triglycerides in very large HDL	XLHDLTG	-0.01 (-0.03 - 0.01)	-0.02 (-0.04 - 0.00)	-0.01 (-0.02 - 0.00)
**Large HDL (average diameter 12.1 nm)**				
Concentration of large HDL particles	LHDLP	0.00 (-0.02 - 0.03)	0.00 (-0.03 - 0.03)	0.00 (-0.01 - 0.01)
Total lipids in large HDL	LHDLL	0.00 (-0.02 - 0.03)	0.00 (-0.03 - 0.03)	0.00 (-0.01 - 0.01)
Phospholipids in large HDL	LHDLPL	0.01 (-0.02 - 0.03)	0.01 (-0.02 - 0.03)	0.00 (-0.01 - 0.01)
Cholesterol in large HDL	LHDLC	0.00 (-0.02 - 0.03)	0.00 (-0.03 - 0.03)	0.00 (-0.01 - 0.01)
Cholesteryl esters in large HDL	LHDLCE	0.00 (-0.02 - 0.03)	0.00 (-0.03 - 0.03)	0.00 (-0.01 - 0.01)
Free cholesterol in large HDL	LHDLFC	0.00 (-0.02 - 0.03)	0.00 (-0.02 - 0.03)	0.00 (-0.01 - 0.01)
Triglycerides in large HDL	LHDLTG	-0.00 (-0.03 - 0.02)	-0.01 (-0.04 - 0.02)	0.00 (-0.01 - 0.01)
**Medium HDL (average diameter 10.9 nm)**				
Concentration of medium HDL particles	MHDLP	0.01 (-0.01 - 0.04)	0.01 (-0.02 - 0.04)	0.00 (-0.01 - 0.01)
Total lipids in medium HDL	MHDLL	0.01 (-0.01 - 0.04)	0.01 (-0.02 - 0.04)	0.00 (-0.01 - 0.01)
Phospholipids in medium HDL	MHDLPL	0.01 (-0.01 - 0.04)	0.01 (-0.02 - 0.04)	0.00 (-0.01 - 0.01)
Cholesterol in medium HDL	MHDLC	0.01 (-0.01 - 0.04)	0.01 (-0.02 - 0.04)	0.01 (-0.00 - 0.02)
Cholesteryl esters in medium HDL	MHDLCE	0.01 (-0.01 - 0.04)	0.02 (-0.02 - 0.05)	0.01 (-0.00 - 0.02)
Free cholesterol in medium HDL	MHDLFC	0.01 (-0.01 - 0.04)	0.01 (-0.02 - 0.04)	0.01 (-0.00 - 0.02)
Triglycerides in medium HDL	MHDLTG	0.00 (-0.02 - 0.02)	-0.00 (-0.03 - 0.02)	-0.01 (-0.01 - 0.00)
**Small HDL (average diameter 8.7 nm)**				
Concentration of small HDL particles	SHDLP	0.00 (-0.02 - 0.02)	0.01 (-0.02 - 0.03)	0.00 (-0.00 - 0.01)
Total lipids in small HDL	SHDLL	0.00 (-0.02 - 0.02)	0.01 (-0.02 - 0.03)	0.00 (-0.00 - 0.01)
Phospholipids in small HDL	SHDLPL	0.01 (-0.02 - 0.03)	0.01 (-0.02 - 0.04)	0.01 (-0.00 - 0.01)
Cholesterol in small HDL	SHDLC	0.00 (-0.02 - 0.02)	0.01 (-0.01 - 0.04)	0.00 (-0.00 - 0.01)
Cholesteryl esters in small HDL	SHDLCE	0.00 (-0.02 - 0.02)	0.01 (-0.01 - 0.04)	0.00 (-0.00 - 0.01)
Free cholesterol in small HDL	SHDLFC	0.01 (-0.02 - 0.03)	0.01 (-0.02 - 0.04)	0.01 (-0.00 - 0.01)
Triglycerides in small HDL	SHDLTG	-0.01 (-0.03 - 0.01)	-0.02 (-0.04 - 0.00)	-0.01 (-0.02 - 0.00)

In the adjusted model for age and sex, differences in the total population between HDL composition and glycocalyx-related parameters were observed. The effect size and 95% confidence intervals are presented. After multiple testing correction, significant associations are highlighted with an asterisk (*).

Table S13. Associations between HDL composition and levels of glycocalyx-related parameters including PBRTotal, PBRfeed vessel, and PBRcapillary in the total population (model 3)

HDL	Abbreviation	PBR total	PBR feed vessel	PBR capillary
**Lipoprotein particle sizes**				
Average diameter for HDL particles	HDLD	0.00 (-0.03 - 0.03)	-0.01 (-0.03 - 0.02)	0.00 (-0.01 - 0.02)
**Lipoprotein particle concentrations**				
Total cholesterol in HDL	HDLC	0.01 (-0.02 - 0.03)	0.01 (-0.02 - 0.04)	0.01 (-0.00 - 0.02)
Total cholesterol in HDL2	HDL2C	0.01 (-0.02 - 0.03)	0.01 (-0.02 - 0.04)	0.01 (-0.00 - 0.02)
Total cholesterol in HDL3	HDL3C	0.00 (-0.02 - 0.03)	0.01 (-0.02 - 0.04)	0.00 (-0.01 - 0.01)
Total triglycerides in HDL	HDLTG	-0.00 (-0.02 - 0.01)	-0.01 (-0.04 - 0.01)	-0.01 (-0.02 - -0.00)
**Apolipoproteins**				
Apolipoprotein A1	ApoA1	0.01 (-0.02 - 0.03)	0.01 (-0.02 - 0.04)	0.00 (-0.01 - 0.01)
**Very large HDL (average diameter 14.3 nm)**				
Concentration of very large HDL particles	XLHDLP	-0.01 (-0.03 - 0.02)	-0.01 (-0.04 - 0.01)	0.00 (-0.01 - 0.01)
Total lipids in very large HDL	XLHDLL	-0.01 (-0.03 - 0.02)	-0.01 (-0.04 - 0.01)	0.00 (-0.01 - 0.01)
Phospholipids in very large HDL	XLHDLPL	-0.01 (-0.03 - 0.02)	-0.01 (-0.04 - 0.02)	0.00 (-0.01 - 0.01)
Cholesterol in very large HDL	XLHDLC	-0.01 (-0.04 - 0.02)	-0.02 (-0.05 - 0.01)	-0.00 (-0.01 - 0.01)
Cholesteryl esters in very large HDL	XLHDLCE	-0.01 (-0.04 - 0.01)	-0.02 (-0.05 - 0.01)	-0.00 (-0.01 - 0.01)
Free cholesterol in very large HDL	XLHDLFC	-0.01 (-0.03 - 0.02)	-0.01 (-0.04 - 0.01)	0.00 (-0.01 - 0.01)
Triglycerides in very large HDL	XLHDLTG	-0.01 (-0.03 - 0.01)	-0.02 (-0.05 - 0.00)	-0.01 (-0.02 - 0.00)
**Large HDL (average diameter 12.1 nm)**				
Concentration of large HDL particles	LHDLP	0.00 (-0.03 - 0.03)	-0.00 (-0.03 - 0.03)	0.00 (-0.01 - 0.02)
Total lipids in large HDL	LHDLL	0.00 (-0.03 - 0.03)	-0.00 (-0.03 - 0.03)	0.00 (-0.01 - 0.02)
Phospholipids in large HDL	LHDLPL	0.00 (-0.02 - 0.03)	0.00 (-0.03 - 0.03)	0.00 (-0.01 - 0.02)
Cholesterol in large HDL	LHDLC	0.00 (-0.03 - 0.03)	-0.00 (-0.03 - 0.03)	0.00 (-0.01 - 0.02)
Cholesteryl esters in large HDL	LHDLCE	0.00 (-0.03 - 0.03)	-0.00 (-0.03 - 0.03)	0.00 (-0.01 - 0.02)
Free cholesterol in large HDL	LHDLFC	0.00 (-0.03 - 0.03)	-0.00 (-0.03 - 0.03)	0.00 (-0.01 - 0.02)
Triglycerides in large HDL	LHDLTG	-0.01 (-0.03 - 0.02)	-0.02 (-0.05 - 0.01)	0.00 (-0.01 - 0.01)
**Medium HDL (average diameter 10.9 nm)**				
Concentration of medium HDL particles	MHDLP	0.01 (-0.01 - 0.04)	0.01 (-0.02 - 0.04)	0.00 (-0.01 - 0.01)
Total lipids in medium HDL	MHDLL	0.01 (-0.01 - 0.04)	0.01 (-0.02 - 0.04)	0.00 (-0.01 - 0.01)
Phospholipids in medium HDL	MHDLPL	0.01 (-0.02 - 0.03)	0.01 (-0.02 - 0.04)	0.00 (-0.01 - 0.01)
Cholesterol in medium HDL	MHDLC	0.01 (-0.01 - 0.04)	0.01 (-0.02 - 0.04)	0.01 (-0.00 - 0.02)
Cholesteryl esters in medium HDL	MHDLCE	0.01 (-0.01 - 0.04)	0.01 (-0.02 - 0.04)	0.01 (-0.00 - 0.02)
Free cholesterol in medium HDL	MHDLFC	0.01 (-0.01 - 0.03)	0.01 (-0.02 - 0.04)	0.01 (-0.00 - 0.02)
Triglycerides in medium HDL	MHDLTG	0.00 (-0.02 - 0.02)	-0.00 (-0.03 - 0.02)	-0.01 (-0.01 - 0.00)
**Small HDL (average diameter 8.7 nm)**				
Concentration of small HDL particles	SHDLP	0.01 (-0.01 - 0.03)	0.01 (-0.02 - 0.04)	0.00 (-0.00 - 0.01)
Total lipids in small HDL	SHDLL	0.01 (-0.01 - 0.03)	0.01 (-0.02 - 0.04)	0.00 (-0.00 - 0.01)
Phospholipids in small HDL	SHDLPL	0.01 (-0.01 - 0.03)	0.01 (-0.02 - 0.04)	0.01 (-0.00 - 0.01)
Cholesterol in small HDL	SHDLC	0.01 (-0.01 - 0.02)	0.01 (-0.01 - 0.04)	0.00 (-0.00 - 0.01)
Cholesteryl esters in small HDL	SHDLCE	0.00 (-0.01 - 0.02)	0.01 (-0.01 - 0.04)	0.00 (-0.00 - 0.01)
Free cholesterol in small HDL	SHDLFC	0.01 (-0.01 - 0.03)	0.01 (-0.02 - 0.04)	0.01 (-0.00 - 0.01)
Triglycerides in small HDL	SHDLTG	-0.01 (-0.03 - 0.01)	-0.02 (-0.04 - 0.00)	-0.01 (-0.02 - 0.00)

In the adjusted model for age, sex, race, menopausal status, lipid-lowering drugs, C-reactive protein, total body fat, and body mass index, differences in the total population between HDL composition and glycocalyx-related parameters were observed. The effect size and 95% confidence intervals are presented. After multiple testing correction, significant associations are highlighted with an asterisk (*).

Table S14. Associations between HDL composition and levels of coagulation parameters including ETP, peak, velocity, lag time and time to peak, FVIII, FIX, FXI, and fibrinogen in women (model 3).

HDL	Abbreviation	ETP	Peak height	Velocity	Lag time	Time to peak	FVIII	FIX	FXI	Fibrinogen
**Lipoprotein particle sizes**										
Average diameter for HDL particles	HDLD	-22.16 (-41.44 - -2.87)*	-2.11 (-4.11 - -0.11	-2.15 (-5.20 - 0.98)	0.24 (0.16 - 0.33)*	0.23 (0.10 - 0.35)*	0.76 (-1.10 - 2.63)	-5.00 (-5.96 - -4.04	-1.67 (-2.85 - -0.49	-4.31 (-6.90 - -1.73)*
**Lipoprotein particle concentrations**										
Total cholesterol in HDL	HDLC	1.87 (-17.04 - 20.78)	0.76 (-1.11 - 2.64)	0.68 (-2.29 - 3.74)	0.19 (0.10 - 0.27)*	0.17 (0.05 - 0.29)*	1.87 (0.09 - 3.65)	-0.43 (-1.43 - 0.56)	1.87 (0.87 - 2.86)*	-3.01 (-5.73 - -0.28)*
Total cholesterol in HDL2	HDL2C	2.47 (-16.45 - 21.38)	0.82 (-1.05 - 2.70)	0.90 (-2.09 - 3.97)	0.20 (0.11 - 0.29)*	0.19 (0.07 - 0.31)*	1.82 (0.04 - 3.60)	-0.65 (-1.65 - 0.35)	1.67 (0.67 - 2.66)*	-3.06 (-5.79 - -0.34)*
Total cholesterol in HDL3	HDL3C	-4.20 (-22.84 - 14.44)	0.02 (-1.78 - 1.83)	-1.47 (-4.22 - 1.35)	0.03 (-0.06 - 0.11)	-0.03 (-0.14 - 0.08)	2.02 (0.27 - 3.76)*	1.68 (0.78 - 2.59)*	3.43 (2.39 - 4.46)*	-1.96 (-4.58 - 0.66)
Total triglycerides in HDL	HDLTG	5.84 (-12.38 - 24.07)	1.90 (0.13 - 3.67)	2.36 (-0.27 - 5.06)	-0.08 (-0.15 - 0.00)	-0.00 (-0.11 - 0.10)	2.00 (0.17 - 3.83)	4.24 (3.24 - 5.24)*	0.82 (-0.20 - 1.83)	-5.70 (-8.34 - -3.07)*
**Apolipoproteins**										
Apolipoprotein A1	ApoA1	-0.38 (-19.02 - 18.25)	0.28 (-1.61 - 2.17)	-0.40 (-3.26 - 2.55)	0.08 (-0.01 - 0.16)	0.04 (-0.08 - 0.15)	2.71 (1.03 - 4.40)*	1.81 (0.86 - 2.75)*	3.32 (2.35 - 4.29)*	-2.93 (-5.58 - -0.28)*
**Very large HDL (average diameter 14.3 nm)**										
Concentration of very large HDL particles	XLHDLP	-24.36 (-41.15 - -7.58)*	-2.40 (-4.26 - -0.53	-2.97 (-5.66 - -0.19	0.14 (0.07 - 0.22)*	0.11 (0.00 - 0.22)	0.90 (-0.72 - 2.53)	-3.39 (-4.26 - -2.52	-0.93 (-1.96 - 0.10)	-3.59 (-5.79 - -1.39)*
Total lipids in very large HDL	XLHDLL	-24.78 (-41.52 - -8.04)*	-2.44 (-4.30 - -0.58	-3.03 (-5.72 - -0.26	0.14 (0.07 - 0.22)*	0.11 (0.00 - 0.22)	0.89 (-0.74 - 2.51)	-3.38 (-4.25 - -2.51	-0.91 (-1.94 - 0.12)	-3.58 (-5.77 - -1.39)*
Phospholipids in very large HDL	XLHDLPL	-19.16 (-36.38 - -1.95)*	-1.79 (-3.69 - 0.12)	-2.05 (-4.84 - 0.82)	0.18 (0.11 - 0.26)*	0.16 (0.05 - 0.27)*	0.84 (-0.83 - 2.50)	-3.60 (-4.49 - -2.70	-1.02 (-2.05 - 0.02)	-3.47 (-5.73 - -1.21)*
Cholesterol in very large HDL	XLHDLC	-30.58 (-46.90 - -14.26)	-3.13 (-4.94 - -1.31	-4.06 (-6.65 - -1.40	0.10 (0.03 - 0.17)*	0.05 (-0.05 - 0.16)	0.84 (-0.75 - 2.43)	-3.20 (-4.04 - -2.36	-0.77 (-1.79 - 0.25)	-3.48 (-5.64 - -1.33)*
Cholesteryl esters in very large HDL	XLHDLCE	-32.01 (-48.33 - -15.68)	-3.37 (-5.19 - -1.55	-4.42 (-6.99 - -1.77	0.09 (0.01 - 0.16)*	0.03 (-0.07 - 0.14)	0.88 (-0.72 - 2.47)	-3.25 (-4.09 - -2.41	-0.78 (-1.81 - 0.24)	-3.36 (-5.52 - -1.19)*
Free cholesterol in very large HDL	XLHDLFC	-26.93 (-43.35 - -10.52)	-2.53 (-4.33 - -0.72	-3.18 (-5.81 - -0.47	0.13 (0.06 - 0.20)*	0.10 (-0.01 - 0.21)	0.75 (-0.85 - 2.36)	-3.05 (-3.90 - -2.20	-0.72 (-1.74 - 0.30)	-3.75 (-5.88 - -1.62)*
Triglycerides in very large HDL	XLHDLTG	-25.55 (-44.10 - -7.01)*	-2.52 (-4.47 - -0.57	-3.47 (-6.25 - -0.60	-0.02 (-0.10 - 0.05)	-0.05 (-0.16 - 0.07)	1.95 (0.24 - 3.66)*	-0.34 (-1.33 - 0.65)	-0.59 (-1.64 - 0.46)	-5.02 (-7.68 - -2.36)*
**Large HDL (average diameter 12.1 nm)**										
Concentration of large HDL particles	LHDLP	-5.52 (-24.03 - 13.00)	0.20 (-1.59 - 2.00)	0.51 (-2.34 - 3.44)	0.26 (0.18 - 0.34)*	0.27 (0.16 - 0.38)*	1.03 (-0.74 - 2.81)	-2.40 (-3.32 - -1.48	-0.47 (-1.52 - 0.57)	-4.40 (-6.83 - -1.98)*
Total lipids in large HDL	LHDLL	-5.74 (-24.18 - 12.69)	0.16 (-1.63 - 1.96)	0.47 (-2.37 - 3.39)	0.26 (0.18 - 0.34)*	0.27 (0.16 - 0.38)*	1.00 (-0.77 - 2.77)	-2.45 (-3.37 - -1.54	-0.50 (-1.54 - 0.54)	-4.37 (-6.79 - -1.96)*
Phospholipids in large HDL	LHDLPL	-3.14 (-22.21 - 15.93)	0.60 (-1.23 - 2.43)	0.93 (-1.98 - 3.92)	0.26 (0.18 - 0.34)*	0.28 (0.16 - 0.39)*	1.23 (-0.60 - 3.05)	-1.85 (-2.79 - -0.91	0.01 (-1.06 - 1.08)	-4.32 (-6.83 - -1.80)*
Cholesterol in large HDL	LHDLC	-7.62 (-25.51 - 10.26)	-0.16 (-1.91 - 1.60)	0.13 (-2.65 - 2.99)	0.25 (0.18 - 0.33)*	0.26 (0.15 - 0.37)*	0.76 (-0.96 - 2.48)	-2.90 (-3.80 - -2.00	-0.83 (-1.85 - 0.19)	-4.29 (-6.64 - -1.94)*
Cholesteryl esters in large HDL	LHDLCE	-7.58 (-25.50 - 10.35)	-0.15 (-1.91 - 1.61)	0.15 (-2.63 - 3.01)	0.26 (0.18 - 0.33)*	0.26 (0.15 - 0.37)*	0.76 (-0.97 - 2.49)	-2.91 (-3.81 - -2.01	-0.85 (-1.87 - 0.17)	-4.33 (-6.68 - -1.98)*
Free cholesterol in large HDL	LHDLFC	-7.76 (-25.53 - 10.00)	-0.17 (-1.91 - 1.57)	0.07 (-2.70 - 2.91)	0.25 (0.17 - 0.32)*	0.25 (0.14 - 0.36)*	0.76 (-0.95 - 2.47)	-2.88 (-3.77 - -1.99	-0.76 (-1.77 - 0.25)	-4.16 (-6.51 - -1.82)*
Triglycerides in large HDL	LHDLTG	-6.84 (-25.11 - 11.44)	-0.26 (-2.10 - 1.57)	-0.12 (-2.91 - 2.74)	0.18 (0.11 - 0.26)*	0.19 (0.09 - 0.30)*	1.55 (-0.09 - 3.20)	-2.19 (-3.10 - -1.29	-1.49 (-2.53 - -0.45	-5.02 (-7.30 - -2.74)*
**Medium HDL (average diameter 10.9 nm)**										
Concentration of medium HDL particles	MHDLP	32.44 (15.19 - 49.70)*	4.77 (3.07 - 6.47)*	6.17 (3.50 - 8.91)*	0.14 (0.06 - 0.22)*	0.21 (0.10 - 0.32)*	2.10 (0.51 - 3.69)*	4.49 (3.53 - 5.46)*	3.85 (2.80 - 4.90)*	-3.85 (-6.36 - -1.35)*
Total lipids in medium HDL	MHDLL	32.00 (14.75 - 49.25)*	4.67 (2.98 - 6.37)*	6.03 (3.37 - 8.77)*	0.14 (0.06 - 0.22)*	0.21 (0.10 - 0.32)*	2.06 (0.46 - 3.65)*	4.33 (3.36 - 5.29)*	3.82 (2.77 - 4.87)*	-3.74 (-6.26 - -1.23)*
Phospholipids in medium HDL	MHDLPL	31.64 (14.03 - 49.25)*	4.84 (3.10 - 6.57)*	6.21 (3.49 - 9.01)*	0.14 (0.06 - 0.22)*	0.22 (0.11 - 0.33)*	2.11 (0.50 - 3.73)*	4.39 (3.44 - 5.33)*	3.66 (2.63 - 4.69)*	-4.26 (-6.75 - -1.77)*
Cholesterol in medium HDL	MHDLC	29.77 (12.79 - 46.76)*	4.08 (2.45 - 5.71)*	5.26 (2.68 - 7.91)*	0.16 (0.07 - 0.24)*	0.20 (0.09 - 0.32)*	1.76 (0.15 - 3.37)	3.51 (2.54 - 4.47)*	3.68 (2.62 - 4.73)*	-2.91 (-5.46 - -0.36)*
Cholesteryl esters in medium HDL	MHDLCE	29.74 (12.90 - 46.58)*	4.01 (2.39 - 5.62)*	5.22 (2.66 - 7.83)*	0.16 (0.07 - 0.24)*	0.20 (0.09 - 0.31)*	1.69 (0.09 - 3.29)	3.42 (2.45 - 4.39)*	3.64 (2.58 - 4.70)*	-2.77 (-5.32 - -0.21)
Free cholesterol in medium HDL	MHDLFC	29.38 (11.83 - 46.92)*	4.28 (2.58 - 5.98)*	5.34 (2.67 - 8.09)*	0.15 (0.07 - 0.23)*	0.20 (0.09 - 0.31)*	1.99 (0.36 - 3.62)*	3.78 (2.83 - 4.73)*	3.75 (2.72 - 4.79)*	-3.39 (-5.92 - -0.85)*
Triglycerides in medium HDL	MHDLTG	21.82 (4.04 - 39.60)*	3.90 (2.05 - 5.74)*	5.21 (2.47 - 8.03)*	-0.10 (-0.17 - -0.03	0.01 (-0.09 - 0.11)	2.11 (0.31 - 3.91)*	6.51 (5.38 - 7.65)*	2.38 (1.31 - 3.46)*	-3.49 (-6.04 - -0.94)*
**Small HDL (average diameter 8.7 nm)**										
Concentration of small HDL particles	SHDLP	34.81 (19.10 - 50.52)*	4.51 (2.90 - 6.12)*	5.22 (2.99 - 7.50)*	0.00 (-0.07 - 0.07)	0.04 (-0.05 - 0.14)	1.82 (0.40 - 3.23)*	5.30 (3.86 - 6.75)*	3.99 (2.78 - 5.20)*	0.14 (-2.09 - 2.38)
Total lipids in small HDL	SHDLL	34.00 (18.30 - 49.70)*	4.37 (2.78 - 5.96)*	5.01 (2.81 - 7.25)*	0.00 (-0.07 - 0.08)	0.04 (-0.05 - 0.13)	1.79 (0.37 - 3.22)*	5.13 (3.72 - 6.53)*	3.98 (2.77 - 5.19)*	0.25 (-1.99 - 2.50)
Phospholipids in small HDL	SHDLPL	45.46 (28.69 - 62.22)*	6.46 (4.63 - 8.29)*	8.79 (6.03 - 11.63)	0.11 (0.04 - 0.18)*	0.23 (0.13 - 0.33)*	1.61 (0.17 - 3.05)*	5.92 (4.41 - 7.43)*	3.52 (2.38 - 4.67)*	-2.11 (-4.53 - 0.31)
Cholesterol in small HDL	SHDLC	19.33 (2.73 - 35.92)*	1.82 (0.38 - 3.25)*	0.99 (-1.07 - 3.09)	-0.07 (-0.14 - 0.00)	-0.11 (-0.20 - -0.03	1.69 (0.09 - 3.29)	3.30 (2.29 - 4.31)*	3.89 (2.67 - 5.10)*	2.58 (0.07 - 5.09)
Cholesteryl esters in small HDL	SHDLCE	14.99 (-2.23 - 32.20)	1.04 (-0.42 - 2.51)	-0.20 (-2.36 - 2.01)	-0.10 (-0.17 - -0.03	-0.17 (-0.26 - -0.08	1.67 (0.01 - 3.34)	2.79 (1.85 - 3.72)*	3.83 (2.62 - 5.03)*	3.40 (0.78 - 6.02)*
Free cholesterol in small HDL	SHDLFC	36.93 (20.87 - 52.98)*	5.24 (3.55 - 6.93)*	6.71 (4.25 - 9.23)*	0.08 (0.00 - 0.15)	0.15 (0.05 - 0.25)*	1.48 (0.02 - 2.94)	5.18 (3.82 - 6.55)*	3.49 (2.38 - 4.61)*	-1.84 (-4.22 - 0.55)
Triglycerides in small HDL	SHDLTG	17.05 (-2.50 - 36.61)	3.21 (1.25 - 5.18)*	3.52 (0.54 - 6.58)*	-0.20 (-0.29 - -0.12	-0.12 (-0.25 - -0.00	1.17 (-0.63 - 2.97)	6.50 (5.39 - 7.62)*	1.73 (0.65 - 2.80)*	-2.17 (-5.26 - 0.91)

In the adjusted model for age, race, menopausal status, lipid-lowering drugs, C-reactive protein, total body fat, and body mass index, differences in the total population between HDL composition and levels of coagulation parameters were observed. The effect size and 95% confidence intervals are presented. After multiple testing correction, significant associations are highlighted with an asterisk (*).

Table S15. Associations between HDL composition and levels of coagulation parameters including ETP, peak, velocity, lag time and time to peak, FVIII, FIX, FXI, and fibrinogen in men (model 3).

HDL	Abbreviation	ETP	Peak height	Velocity	Lag time	Time to peak	FVIII	FIX	FXI	Fibrinogen
**Lipoprotein particle sizes**										
Average diameter for HDL particles	HDLD	-11.75 (-32.17 - 8.68)	-1.07 (-2.89 - 0.75)	-1.86 (-4.78 - 1.15)	0.35 (0.24 - 0.46)*	0.30 (0.17 - 0.44)*	-2.23 (-4.14 - -0.32	-3.85 (-4.81 - -2.89	-1.99 (-3.10 - -0.88	-6.81 (-10.00 - -3.62)*
**Lipoprotein particle concentrations**										
Total cholesterol in HDL	HDLC	-10.42 (-30.97 - 10.13)	-0.56 (-2.50 - 1.37)	-1.85 (-4.92 - 1.31)	0.30 (0.19 - 0.41)*	0.23 (0.09 - 0.37)*	-2.08 (-3.97 - -0.20	-1.40 (-2.32 - -0.48	0.06 (-1.07 - 1.19)	-5.73 (-8.90 - -2.56)*
Total cholesterol in HDL2	HDL2C	-9.68 (-30.27 - 10.91)	-0.46 (-2.39 - 1.47)	-1.61 (-4.68 - 1.56)	0.32 (0.21 - 0.42)*	0.25 (0.11 - 0.39)*	-2.05 (-3.95 - -0.15	-1.56 (-2.49 - -0.63	-0.15 (-1.29 - 0.99)	-6.06 (-9.24 - -2.87)*
Total cholesterol in HDL3	HDL3C	-14.39 (-33.35 - 4.58)	-1.25 (-3.12 - 0.63)	-3.51 (-6.40 - -0.52	0.09 (-0.01 - 0.18)	-0.03 (-0.16 - 0.11)	-1.92 (-3.57 - -0.27	0.21 (-0.62 - 1.05)	1.82 (0.77 - 2.88)*	-1.84 (-4.65 - 0.98)
Total triglycerides in HDL	HDLTG	-5.57 (-21.07 - 9.93)	0.06 (-1.38 - 1.51)	0.14 (-2.17 - 2.51)	-0.22 (-0.31 - -0.14	-0.21 (-0.31 - -0.10	1.23 (-0.53 - 2.99)	3.93 (3.06 - 4.81)*	1.61 (0.69 - 2.54)*	-2.04 (-4.09 - -0.00)
**Apolipoproteins**										
Apolipoprotein A1	ApoA1	-15.06 (-35.47 - 5.36)	-1.12 (-3.07 - 0.84)	-3.39 (-6.44 - -0.24	0.11 (0.00 - 0.22)	-0.01 (-0.15 - 0.14)	-1.48 (-3.40 - 0.43)	1.52 (0.56 - 2.48)*	2.31 (1.12 - 3.50)*	-5.94 (-9.07 - -2.81)*
**Very large HDL (average diameter 14.3 nm)**										
Concentration of very large HDL particles	XLHDLP	-19.53 (-42.99 - 3.92)	-2.04 (-4.13 - 0.05)	-3.45 (-6.65 - -0.14	0.25 (0.12 - 0.37)*	0.17 (0.01 - 0.32)	-2.15 (-4.31 - 0.01)	-3.33 (-4.41 - -2.26	-1.61 (-2.88 - -0.34	-5.45 (-8.89 - -2.01)*
Total lipids in very large HDL	XLHDLL	-20.29 (-43.77 - 3.20)	-2.10 (-4.19 - -0.01	-3.52 (-6.72 - -0.21	0.24 (0.11 - 0.37)*	0.16 (0.01 - 0.32)	-2.18 (-4.34 - -0.02	-3.33 (-4.40 - -2.25	-1.58 (-2.85 - -0.31	-5.43 (-8.88 - -1.99)*
Phospholipids in very large HDL	XLHDLPL	-13.48 (-36.72 - 9.76)	-1.28 (-3.36 - 0.79)	-2.08 (-5.36 - 1.31)	0.32 (0.19 - 0.45)*	0.27 (0.11 - 0.43)*	-1.94 (-4.06 - 0.18)	-3.68 (-4.76 - -2.60	-1.99 (-3.25 - -0.74	-6.05 (-9.46 - -2.65)*
Cholesterol in very large HDL	XLHDLC	-26.18 (-49.28 - -3.09)	-2.83 (-4.88 - -0.78	-4.75 (-7.80 - -1.59	0.15 (0.03 - 0.27)*	0.05 (-0.10 - 0.20)	-2.38 (-4.50 - -0.26	-2.90 (-3.94 - -1.86	-1.03 (-2.29 - 0.23)	-4.28 (-7.67 - -0.89)*
Cholesteryl esters in very large HDL	XLHDLCE	-24.40 (-47.13 - -1.67)	-2.83 (-4.85 - -0.81	-4.90 (-7.89 - -1.81	0.13 (0.00 - 0.25)	0.01 (-0.13 - 0.16)	-2.34 (-4.44 - -0.24	-2.87 (-3.89 - -1.85	-0.91 (-2.15 - 0.33)	-3.54 (-6.88 - -0.19)
Free cholesterol in very large HDL	XLHDLFC	-29.48 (-53.06 - -5.90)*	-2.70 (-4.79 - -0.61	-4.15 (-7.34 - -0.85	0.21 (0.07 - 0.34)*	0.14 (-0.02 - 0.30)	-2.37 (-4.51 - -0.23	-2.86 (-3.93 - -1.78	-1.29 (-2.58 - -0.01	-5.95 (-9.43 - -2.48)*
Triglycerides in very large HDL	XLHDLTG	-15.99 (-32.35 - 0.37)	-1.83 (-3.30 - -0.37	-3.68 (-5.92 - -1.39	-0.14 (-0.22 - -0.06	-0.22 (-0.32 - -0.13	-0.31 (-1.89 - 1.27)	1.03 (0.11 - 1.96)	0.20 (-0.86 - 1.25)	-1.43 (-3.90 - 1.05)
**Large HDL (average diameter 12.1 nm)**										
Concentration of large HDL particles	LHDLP	-2.80 (-23.50 - 17.91)	-0.28 (-2.15 - 1.59)	-1.04 (-4.09 - 2.10)	0.39 (0.27 - 0.50)*	0.33 (0.19 - 0.47)*	-1.37 (-3.34 - 0.60)	-2.62 (-3.66 - -1.58	-1.51 (-2.64 - -0.39	-7.29 (-10.47 - -4.12)*
Total lipids in large HDL	LHDLL	-3.13 (-23.95 - 17.69)	-0.30 (-2.18 - 1.58)	-1.04 (-4.10 - 2.12)	0.39 (0.27 - 0.50)*	0.33 (0.19 - 0.48)*	-1.40 (-3.37 - 0.58)	-2.67 (-3.72 - -1.63	-1.55 (-2.68 - -0.41	-7.34 (-10.53 - -4.15)*
Phospholipids in large HDL	LHDLPL	-1.39 (-21.25 - 18.48)	-0.19 (-2.00 - 1.61)	-1.05 (-3.99 - 1.99)	0.37 (0.26 - 0.48)*	0.30 (0.17 - 0.44)*	-1.21 (-3.13 - 0.71)	-2.13 (-3.14 - -1.12	-1.05 (-2.13 - 0.03)	-6.70 (-9.75 - -3.65)*
Cholesterol in large HDL	LHDLC	-5.31 (-26.90 - 16.28)	-0.42 (-2.36 - 1.52)	-0.99 (-4.15 - 2.27)	0.40 (0.28 - 0.52)*	0.36 (0.21 - 0.51)*	-1.57 (-3.59 - 0.44)	-3.14 (-4.21 - -2.07	-1.94 (-3.12 - -0.75	-7.80 (-11.09 - -4.50)*
Cholesteryl esters in large HDL	LHDLCE	-5.21 (-26.72 - 16.29)	-0.43 (-2.36 - 1.51)	-1.01 (-4.15 - 2.24)	0.40 (0.28 - 0.52)*	0.36 (0.21 - 0.51)*	-1.56 (-3.57 - 0.45)	-3.14 (-4.21 - -2.07	-1.97 (-3.15 - -0.79	-7.83 (-11.12 - -4.54)*
Free cholesterol in large HDL	LHDLFC	-5.58 (-27.40 - 16.24)	-0.39 (-2.35 - 1.56)	-0.92 (-4.11 - 2.38)	0.40 (0.27 - 0.52)*	0.35 (0.20 - 0.50)*	-1.61 (-3.63 - 0.42)	-3.13 (-4.20 - -2.06	-1.82 (-3.03 - -0.62	-7.66 (-10.98 - -4.35)*
Triglycerides in large HDL	LHDLTG	5.22 (-14.28 - 24.72)	0.13 (-1.66 - 1.92)	-0.94 (-3.82 - 2.04)	0.26 (0.16 - 0.36)*	0.19 (0.06 - 0.32)*	-0.73 (-2.61 - 1.15)	-2.03 (-3.03 - -1.03	-1.93 (-3.01 - -0.85	-5.70 (-8.81 - -2.59)*
**Medium HDL (average diameter 10.9 nm)**										
Concentration of medium HDL particles	MHDLP	-8.04 (-25.62 - 9.53)	0.50 (-1.21 - 2.20)	0.13 (-2.68 - 3.02)	0.16 (0.06 - 0.25)*	0.16 (0.03 - 0.28)*	0.21 (-1.48 - 1.90)	2.85 (1.98 - 3.72)*	1.63 (0.54 - 2.72)*	-7.23 (-9.97 - -4.49)*
Total lipids in medium HDL	MHDLL	-7.97 (-25.70 - 9.75)	0.47 (-1.26 - 2.20)	0.07 (-2.76 - 2.99)	0.17 (0.07 - 0.26)*	0.17 (0.04 - 0.29)*	0.11 (-1.58 - 1.80)	2.63 (1.76 - 3.51)*	1.54 (0.45 - 2.63)*	-7.19 (-9.95 - -4.43)*
Phospholipids in medium HDL	MHDLPL	-8.02 (-25.61 - 9.58)	0.57 (-1.13 - 2.27)	0.33 (-2.48 - 3.23)	0.16 (0.06 - 0.25)*	0.16 (0.04 - 0.29)*	0.11 (-1.59 - 1.81)	2.77 (1.89 - 3.64)*	1.50 (0.42 - 2.58)*	-7.02 (-9.73 - -4.31)*
Cholesterol in medium HDL	MHDLC	-7.49 (-25.70 - 10.71)	0.30 (-1.51 - 2.10)	-0.27 (-3.18 - 2.73)	0.21 (0.12 - 0.31)*	0.20 (0.07 - 0.32)*	-0.24 (-1.90 - 1.42)	1.64 (0.77 - 2.51)*	1.14 (0.05 - 2.24)	-6.95 (-9.80 - -4.10)*
Cholesteryl esters in medium HDL	MHDLCE	-7.57 (-25.75 - 10.60)	0.27 (-1.53 - 2.07)	-0.29 (-3.19 - 2.70)	0.21 (0.12 - 0.31)*	0.20 (0.08 - 0.32)*	-0.22 (-1.87 - 1.44)	1.60 (0.74 - 2.47)*	1.11 (0.01 - 2.21)	-7.03 (-9.88 - -4.18)*
Free cholesterol in medium HDL	MHDLFC	-7.07 (-25.33 - 11.19)	0.38 (-1.41 - 2.18)	-0.19 (-3.11 - 2.82)	0.20 (0.11 - 0.30)*	0.19 (0.06 - 0.31)*	-0.31 (-2.01 - 1.39)	1.76 (0.87 - 2.65)*	1.25 (0.15 - 2.34)	-6.51 (-9.37 - -3.65)*
Triglycerides in medium HDL	MHDLTG	-1.64 (-17.47 - 14.18)	0.53 (-0.97 - 2.02)	0.88 (-1.51 - 3.33)	-0.23 (-0.31 - -0.16	-0.20 (-0.31 - -0.10	2.14 (0.45 - 3.83)*	5.08 (4.29 - 5.86)*	2.42 (1.55 - 3.29)*	-1.35 (-3.44 - 0.75)
**Small HDL (average diameter 8.7 nm)**										
Concentration of small HDL particles	SHDLP	-0.57 (-18.01 - 16.88)	0.98 (-0.86 - 2.83)	0.63 (-2.34 - 3.70)	0.01 (-0.08 - 0.10)	0.00 (-0.13 - 0.13)	0.23 (-1.28 - 1.75)	3.72 (2.48 - 4.97)*	2.66 (1.44 - 3.89)*	-1.79 (-4.29 - 0.72)
Total lipids in small HDL	SHDLL	-0.35 (-17.82 - 17.12)	0.94 (-0.91 - 2.80)	0.53 (-2.46 - 3.60)	0.02 (-0.06 - 0.11)	0.01 (-0.12 - 0.14)	0.13 (-1.37 - 1.62)	3.43 (2.21 - 4.65)*	2.57 (1.34 - 3.79)*	-1.67 (-4.16 - 0.82)
Phospholipids in small HDL	SHDLPL	-7.46 (-24.64 - 9.73)	1.00 (-0.69 - 2.68)	1.69 (-1.05 - 4.51)	0.05 (-0.04 - 0.14)	0.12 (-0.00 - 0.24)	0.69 (-1.06 - 2.44)	4.54 (3.52 - 5.56)*	1.67 (0.60 - 2.74)*	-5.85 (-8.55 - -3.14)*
Cholesterol in small HDL	SHDLC	5.28 (-11.16 - 21.72)	0.56 (-1.23 - 2.34)	-0.56 (-3.41 - 2.38)	0.04 (-0.03 - 0.12)	-0.03 (-0.15 - 0.10)	-0.47 (-1.83 - 0.89)	0.88 (0.03 - 1.72)	2.03 (0.87 - 3.18)*	1.56 (-0.54 - 3.67)
Cholesteryl esters in small HDL	SHDLCE	7.36 (-8.74 - 23.46)	0.52 (-1.21 - 2.25)	-0.69 (-3.45 - 2.15)	0.04 (-0.04 - 0.11)	-0.04 (-0.16 - 0.08)	-0.44 (-1.83 - 0.95)	0.41 (-0.36 - 1.18)	1.92 (0.81 - 3.04)*	2.40 (0.35 - 4.46)*
Free cholesterol in small HDL	SHDLFC	-11.72 (-29.93 - 6.48)	0.54 (-1.32 - 2.39)	0.63 (-2.32 - 3.67)	0.04 (-0.05 - 0.13)	0.08 (-0.04 - 0.21)	-0.46 (-2.07 - 1.16)	3.71 (2.55 - 4.87)*	1.81 (0.70 - 2.93)*	-5.05 (-7.75 - -2.34)*
Triglycerides in small HDL	SHDLTG	-4.39 (-20.43 - 11.66)	0.34 (-1.12 - 1.80)	0.83 (-1.54 - 3.24)	-0.30 (-0.39 - -0.20	-0.25 (-0.37 - -0.13	1.06 (-0.81 - 2.93)	4.55 (3.57 - 5.53)*	2.22 (1.28 - 3.15)*	0.71 (-1.63 - 3.05)

In the adjusted model for age, race, lipid-lowering drugs, C-reactive protein, total body fat, and body mass index, differences in the total population between HDL composition and levels of coagulation parameters were observed. The effect size and 95% confidence intervals are presented. After multiple testing correction, significant associations are highlighted with an asterisk (*).
